# A high spatial resolution land surface phenology dataset for AmeriFlux and NEON sites

**DOI:** 10.1038/s41597-022-01570-5

**Published:** 2022-07-27

**Authors:** Minkyu Moon, Andrew D. Richardson, Thomas Milliman, Mark A. Friedl

**Affiliations:** 1 Department of Earth and Environment, Boston University, Boston, USA; 2 School of Informatics, Computing & Cyber Systems, Northern Arizona University, Flagstaff, USA; 3 Center for Ecosystem Science and Society, Northern Arizona University, Flagstaff, USA; 4 Earth Systems Research Center, University of New Hampshire, Durham, USA

**Keywords:** Biogeography, Macroecology

## Abstract

Vegetation phenology is a key control on water, energy, and carbon fluxes in terrestrial ecosystems. Because vegetation canopies are heterogeneous, spatially explicit information related to seasonality in vegetation activity provides valuable information for studies that use eddy covariance measurements to study ecosystem function and land-atmosphere interactions. Here we present a land surface phenology (LSP) dataset derived at 3 m spatial resolution from PlanetScope imagery across a range of plant functional types and climates in North America. The dataset provides spatially explicit information related to the timing of phenophase changes such as the start, peak, and end of vegetation activity, along with vegetation index metrics and associated quality assurance flags for the growing seasons of 2017–2021 for 10 × 10 km windows centred over 104 eddy covariance towers at AmeriFlux and National Ecological Observatory Network (NEON) sites. These LSP data can be used to analyse processes controlling the seasonality of ecosystem-scale carbon, water, and energy fluxes, to evaluate predictions from land surface models, and to assess satellite-based LSP products.

## Abstract (summary)

**Table:** 

Measurement(s)	vegetation phenology
Technology Type(s)	satellite imagery

## Background & Summary

The AmeriFlux network^1^, which is part of the global FLUXNET^2^ network of eddy covariance towers, is an important tool for measuring land-atmosphere exchanges of carbon, energy, and water at local to global scales^3,4^. The network uses eddy covariance instruments and standardized data processing techniques to provide non-destructive measurements of ecosystem fluxes at high temporal resolution, which makes it a unique and powerful tool for studying how ecosystems are influencing and responding to climate change^1–4^. However, analysing and interpreting how measured fluxes are affected by local environmental conditions, and particularly spatio-temporal variation in local vegetation properties, is challenging. Many eddy covariance towers now have PhenoCams^5,6^, which provide valuable information and imagery that can be used to characterize and monitor canopy conditions in the vicinity of towers. However, PhenoCams have fixed and limited fields of view that only capture local conditions in the camera field of view or region of interest. Given the important role of vegetation phenology in controlling fluxes of carbon, water, and energy, spatially explicit information related to vegetation phenology has significant utility for studies that use eddy covariance tower data to quantify and interpret land-atmosphere interactions and their role in ecosystem function and weather and climate processes^7–10^.

Remote sensing has been used for several decades to monitor and map the land surface phenology (LSP) of terrestrial ecosystems^11–14^ and has also been used to estimate models of land-atmosphere fluxes calibrated to eddy covariance measurements^15–18^. Until recently, however, the temporal frequency of image acquisitions required for this latter application has constrained these studies to using coarse spatial resolution imagery (e.g., MODIS at 500 m), which limits their utility for understanding how local variability in landscape properties influence fluxes. Bolton *et al*.^19^ recently demonstrated that harmonized Landsat and Sentinel-2 data can provide high-quality LSP information at 30 m spatial resolution, but even this resolution fails to capture fine-scale variation (i.e., below 30 m) in phenology and vegetation cover (related to, for example, species composition or patchy vegetation structure) that can influence land-atmosphere fluxes^20–22^.

Several recent studies have demonstrated that commercially available PlanetScope imagery can be used to monitor and map LSP at fine spatial resolution, thereby providing new opportunities to exploit high spatial resolution LSP measurements for studies that require spatially explicit information related to vegetation phenology^23–26^. PlanetScope imagery is acquired by a constellation of CubeSats (180 + as of 2022), and provides daily imagery in four bands spanning the visible and near-infrared wavelengths at a nominal spatial resolution of 3 m^27^. Although PlanetScope data do not have the radiometric fidelity and geometric accuracy^28,29^ of publicly available medium spatial resolution imagery from Landsat 8 and Sentinel-2, the higher spatial and temporal resolution of PlanetScope imagery creates new opportunities to investigate a wide array of land surface properties and processes. For example, Moon *et al*.^26^ used PlanetScope imagery in association with the LSP algorithm developed by Bolton *et al*.^19^ and reported that LSP metrics estimated using PlanetScope imagery show strong agreement with LSP metrics derived from high-quality publicly available 30 m satellite imagery (i.e., Landsat 8 and Sentinel 2) and from PhenoCams. Their results demonstrated that PlanetScope imagery captures useful information related to vegetation phenology at 3 m spatial resolution across a range of ecosystem types^26^, and that fine-scale variation in landscape properties arising from land use, water bodies, species composition, and vegetation structure results in substantial spatial variation in phenology that is not captured at medium spatial resolution (i.e., 30 m).

**Fig. 1:**
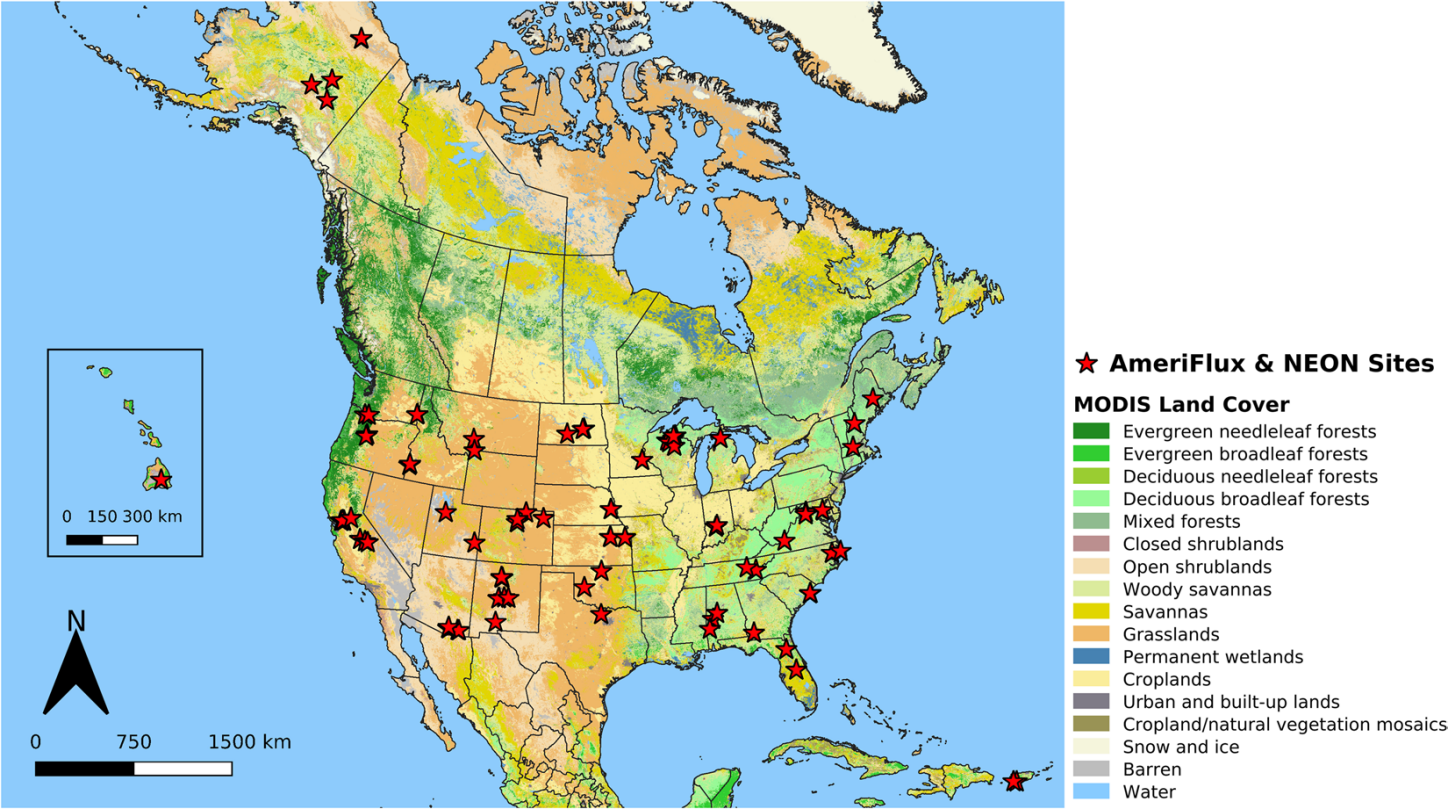
Geographic distribution of sites included in the PLSP dataset across North America. Each site includes data from a 10 × 10 km square centred over one AmeriFlux or NEON site (n = 104). Note that several adjacent sites are not distinguishable on the map. The background map shows the IGBP land cover type from the MODIS Land Cover Type product^35^.

Building on the proof-of-concept provided by Moon *et al*.^26^, here we present a new LSP dataset derived from 3 m spatial resolution PlanetScope imagery covering 104 AmeriFlux and NEON sites that encompass a wide range of plant functional types, biomes, and climates regimes in North America (Fig. 1). The dataset includes standard LSP metrics that are commonly derived from remote sensing such as the day of year corresponding to the start, peak, and end of vegetation greenness, along with metrics that provide the maximum, amplitude, and growing season sum of the two-band Enhanced Vegetation Index^30^ (EVI2) and associated quality assurance flags. Data for the period 2017 to 2021 for areas that encompass the flux footprint and surrounding landscapes at each tower site (10 × 10 km = 10^8^ m^2^) are included in the dataset. To document and illustrate the quality of these data, we present results from a comprehensive assessment using two independent data sources: (1) LSP metrics from the Multisensor Land Surface Phenology dataset, which is derived from Harmonized Landsat 8 and Sentinel-2 imagery^31^; and (2) phenometrics from the PhenoCam V3 Dataset.

## Methods

### Site selection

We selected 104 sites covering a range of ecological, land cover, and climate conditions across North America (Table 1). These sites were selected because they are part of either the National Ecological Observatory Network (NEON) or AmeriFlux network, all have PhenoCams, and each has at least one year of available flux data between 2017 and 2021. Among the included sites, 44 are part of the NEON.

**Table 1:** List of AmeriFlux and NEON sites included in the dataset. .

Site Code	Site Full Name	Site Code	Site Full Name
PR-xGU	NEON Guanica Forest	US-Var	Vaira Ranch
PR-xLA	NEON Lajas Experimental Station	US-Vcm	Valles Caldera Mixed Conifer
US-ALQ	Allequash Creek Site	US-Vcp	Valles Caldera Ponderosa Pine
US-ARM	ARM Southern Great Plains site	US-Vcs	Valles Caldera Sulphur Springs Mixed Conifer
US-Bi1	Bouldin Island Alfalfa	US-WCr	Willow Creek
US-Bi2	Bouldin Island corn	US-Whs	Walnut Gulch Lucky Hills Shrub
US-BMM	Bangtail Mountain Meadow	US-Wjs	Willard Juniper Savannah
US-BRG	Bayles Road Grassland Tower	US-Wkg	Walnut Gulch Kendall Grasslands
US-CF1	CAF-LTAR Cook East	US-xAB	NEON Abby Road
US-CF2	CAF-LTAR Cook West	US-xAE	NEON Klemme Range Research Station
US-CF3	CAF-LTAR Boyd North	US-xBL	NEON Blandy Experimental Farm
US-CF4	CAF-LTAR Boyd South	US-xBN	NEON Caribou Creek - Poker Flats Watershed
US-Ha1	Harvard Forest EMS Tower	US-xBR	NEON Bartlett Experimental Forest
US-Ha2	Harvard Forest Hemlock Site	US-xCL	NEON LBJ National Grassland
US-HB1	North Inlet Crab Haul Creek	US-xCP	NEON Central Plains Experimental Range
US-HB2	Hobcaw Barony Mature Longleaf Pine	US-xDC	NEON Dakota Coteau Field School
US-HB3	Hobcaw Barony Longleaf Pine Restoration	US-xDJ	NEON Delta Junction
US-Ho1	Howland Forest	US-xDL	NEON Dead Lake
US-ICs	Imnavait Creek Watershed Wet Sedge Tundra	US-xDS	NEON Disney Wilderness Preserve
US-KFS	Kansas Field Station	US-xGR	NEON Great Smoky Mountains National Park
US-Me2	Metolius mature ponderosa pine	US-xHA	NEON Harvard Forest
US-Me6	Metolius Young Pine Burn	US-xHE	NEON Healy
US-MMS	Morgan Monroe State Forest	US-xJE	NEON Jones Ecological Research Center
US-Mpj	Mountainair Pinyon-Juniper Woodland	US-xJR	NEON Jornada LTER
US-Myb	Mayberry Wetland	US-xKA	NEON Konza Prairie Biological Station-Relocatable
US-NC2	NC_Loblolly Plantation	US-xKZ	NEON Konza Prairie Biological Station
US-NC3	NC_Clearcut#3	US-xLE	NEON Lenoir Landing
US-NC4	NC_AlligatorRiver	US-xMB	NEON Moab
US-Ne1	Mead-irrigated continuous maize site	US-xML	NEON Mountain Lake Biological Station
US-Ne2	Mead-irrigated maize-soybean rotation site	US-xNG	NEON Northern Great Plains Research Laboratory
US-Ne3	Mead-rainfed maize-soybean rotation site	US-xNQ	NEON Onaqui-Ault
US-NR1	Niwot Ridge Forest	US-xNW	NEON Niwot Ridge Mountain Research Station
US-PFa	Park Falls	US-xPU	NEON Pu’u Maka’ala Natural Area Reserve
US-Rms	RCEW Mountain Big Sagebrush	US-xRM	NEON Rocky Mountain National Park
US-Ro4	Rosemount Prairie	US-xRN	NEON Oak Ridge National Lab
US-Ro5	Rosemount I18_South	US-xSB	NEON Ordway-Swisher Biological Station
US-Ro6	Rosemount I18_North	US-xSC	NEON Smithsonian Conservation Biology Institute
US-Rws	Reynolds Creek Wyoming big sagebrush	US-xSE	NEON Smithsonian Environmental Research Center
US-Seg	Sevilleta grassland	US-xSJ	NEON San Joaquin Experimental Range
US-Ses	Sevilleta shrubland	US-xSL	NEON North Sterling
US-Sne	Sherman Island Restored Wetland	US-xSP	NEON Soaproot Saddle
US-Snf	Sherman Barn	US-xSR	NEON Santa Rita Experimental Range
US-SRG	Santa Rita Grassland	US-xST	NEON Steigerwaldt Land Services
US-SRM	Santa Rita Mesquite	US-xTA	NEON Talladega National Forest
US-Syv	Sylvania Wilderness Area	US-xTE	NEON Lower Teakettle
US-Ton	Tonzi Ranch	US-xTL	NEON Toolik
US-Tw1	Twitchell Wetland West Pond	US-xTR	NEON Treehaven
US-Tw3	Twitchell Alfalfa	US-xUK	NEON The University of Kansas Field Station
US-Tw4	Twitchell East End Wetland	US-xUN	NEON Univ. of Notre Dame Environmental Research Center
US-Tw5	East Pond Wetland	US-xWD	NEON Woodworth
US-UMB	Univ. of Mich. Biological Station	US-xWR	NEON Wind River Experimental Forest
US-UMd	UMBS Disturbance	US-xYE	NEON Yellowstone Northern Range

### PlanetScope image database compilation

The LSP metrics included in the dataset are derived from a database of daily 3 m PlanetScope imagery. To compile this database, a Python script was created to search, request, and download imagery using Planet’s RESTful API interface (https://developers.planet.com/docs/apis/data/). For each site, the area of interest (AOI) was defined using a GeoJSON file that prescribed a 10 by 10 km box centered over the flux tower at each site. Each GeoJSON was then used to submit search requests to the API. As part of the search process, the following filters were applied to ensure that good quality images with adequate clear sky views and high-accuracy geolocation were downloaded: (1) quality category identified as ‘standard’; (2) cloud cover less than or equal to 50%; and (3) ground control is ‘true’. Filtering was performed using all available PlanetScope ‘PSScene4Band’ imagery from 2016 to 2022. Once the API completed the search, the Python script read the search results, submitted orders, and the selected imagery was downloaded from Planet’s cloud-based system to local storage. During execution of the Python script, a log file was created to keep track of successful and failed orders. If an order failed, the script was run again targeting the specific order that failed. The resulting dataset included over 1.8 million unique files with, on average, 3,885 scene images for each site (i.e., the number of images, on average, that overlap part of each 10 by 10 km site), and had a total volume of 62.2 TB.

### Image processing

To ensure that high-quality image time series were used to generate LSP metrics, we used PlanetScope per-pixel quality assurance information to exclude pixels that had low quality in all 4 bands (i.e., blue, green, red, and near-infrared). Specifically, we excluded pixels where the Unusable Data Mask (layer ‘umd’) was not 0 (i.e., we retained pixels that were not cloud contaminated or located in non-image areas) and pixels where the Usable Data Mask (layer ‘umd2’) is 0 (i.e., we retained pixels that were not contaminated by snow, shadow, haze, or clouds). We then cropped all the images to exclude pixels outside of the 10 by 10 km window centered over each tower. We selected this window size based on published results showing that 80% of the average monthly footprint at eddy covariance towers ranges from 10^3^ to 10^7^ square meters^22^. Note that the swath for PlanetScope imagery often did not cover entire sites and some sites (e.g., the tall tower at US-Pfa) have larger footprints than other sites. Similarly, most sites had multiple PlanetScope image acquisitions on the same day. To create image time series, we mosaiced all available imagery at each site on each date, and, under the assumption that geolocation error was non-systematic and modest, we created a single image for each date using the mean surface reflectance for pixels with multiple values on the same day. The resulting database of daily surface reflectance images were sorted in chronological order, sub-divided into 200 sub-areas at each site (i.e., 0.5 km^2^ each), and then stored as image stacks to facilitate parallel processing to estimate LSP metrics, where each image stack included all images from July 1, 2016 through January 31, 2022.

### Creation of daily EVI2 time series

To estimate LSP metrics we adapted the algorithm described by Bolton *et al*.^19^, which was originally implemented to estimate LSP metrics from harmonized Landsat and Sentinel-2 (HLS) imagery, for use with PlanetScope imagery. Prior to LSP estimation, daily images of the two-band Enhanced Vegetation Index^30^ (EVI2) data were generated from PlanetScope imagery and then interpolated to create smooth time series of daily EVI2 values at each pixel in three main steps. First, sources of variation related to clouds, atmospheric aerosols, and snow were detected and removed from the EVI2 time series at each pixel based on data masks provided with PlanetScope imagery (described above) and outlier detection criteria (i.e., de-spiking and removing negative EVI2 values). Second, we identified the ‘background’ EVI2 value (the minimum EVI2 value outside of the growing season) based on the 10^th^ percentile of snow-free EVI2 values at each pixel. Any dates with EVI2 values below the background value were replaced with the background EVI2. Third, penalized cubic smoothing splines were used to gap-fill and smooth the data to create daily EVI2 time series across all years of available data. Complete details on these steps are given in Bolton *et al*.^19^. This approach has been tested and shown to yield PlanetScope EVI2 time series that are consistent with both EVI2 time series from HLS imagery and time series of the Green Chromatic Coordinate (*G*_*CC*_) from PhenoCam imagery^26^. We used the EVI2 instead of other vegetation indices such as the Enhanced Vegetation Index (EVI) or the Normalized Difference Vegetation Index (NDVI) because EVI2 is less sensitive to noise from atmospheric effects relative to EVI and is less prone to saturation over dense canopies and noise from variation in soil background reflectance over sparse canopies relative to the NDVI^30,32^. Thus, phenological metrics from EVI2 time series tend to have better agreement with PhenoCam observations than corresponding metrics from NDVI^33^.

### Identifying phenological cycles

Prior to estimating LSP metrics, we first identity unique growth cycles by searching the period before and after each local peak in the daily PlanetScope EVI2 time series. To be considered a valid growth cycle, the difference in EVI2 between the local minimum and maximum was required to be at least 0.1 and greater than 35% of the total range in EVI2 over the 24-month period centered on the target year ± 6 months. The start of each growth cycle is restricted to occur within 185 days before the peak of the cycle and at least 30 days after the previous peak. The same procedure was applied in reverse at the end of the cycle to constrain the range of end dates for each growth cycle. This procedure is applied recursively over the time series until each local peak has been assessed and all growth cycles (with associated green-up period, peak greenness, and green-down period) are identified in the time series at each pixel. As part of this process, the algorithm provides the number of growth cycles identified for each year in the time series.

### Retrieving LSP metrics

LSP metrics are estimated for each pixel in up to two growth cycles in each year. If no growth cycles are detected, the algorithm returns fill values for all timing metrics, but does report values for the four annual metrics: EVImax, EVIamp, EVIarea, and numObs (see below). If more than two growth cycles are detected, which is rare but does occur (e.g., alfalfa, which is harvested and regrows multiple times in a year), the algorithm records 7 LSP metrics for each of the two growth cycles with the largest EVI2 amplitudes. The resulting dataset includes seven ‘timing’ metrics that identify the timing of greenup onset, mid-greenup, maturity, peak EVI2, greendown onset, mid-greendown, and dormancy. These metrics record the day of year (DOY) when the EVI2 time series exceeds 15%, 50%, and 90% of EVI2 amplitude during the greenup phase, reaches its maximum, and goes below 90%, 50%, and 15% of EVI2 amplitude during the greendown phase. In addition, the algorithm records three complementary metrics that characterize the magnitude of seasonality and total ‘greenness’ at each pixel in each growth cycle: the EVI2 amplitude, the maximum EVI2, and the growing season integral of EVI2, which is calculated as the sum of daily EVI2 values between the growth cycle start- and end-dates (i.e., from greenup onset to dormancy).

### Quality assurance flags

Quality Assurance (QA) values are estimated at each pixel based on the density of observations and the quality of spline fits during each phenophase of the growing season. A QA value of 1 (high quality) is assigned if the correlation between observed versus fitted daily EVI2 values is greater than 0.75 and the maximum gap during each phase is less than 30 days. A QA value of 2 (moderate quality) is assigned if the correlation coefficient is less than 0.75 or the length of the maximum gap over the segment is greater than 30 days. A QA value of 3 (low quality) is assigned if the correlation coefficient is less than 0.75 and the length of the maximum gap over the segment is greater than 30 days. A QA value of 4 is assigned if no growth cycles were detected or insufficient data were available to run the algorithm.

## Data Records

The PlanetScope Land Surface Phenology (PLSP) dataset^34^ consists of 24 data layers (Table 2) and spans 5 years (2017–2021) for each site. Each site and year of data is saved in a single Network Common Data Form (netCDF) file, and the dataset is permanently and publicly available through the Oak Ridge National Lab Distributed Active Archive Centre for Biogeochemical Dynamics (10.3334/ORNLDAAC/2033).

**Table 2:** Product table.

Layer Name	Description	Units	Scale Factor	Valid Range	Fill value
NumCycles	Number of phenological cycles detected in target year	Number of cycles	1	0–6	32767
**First Vegetation Cycle: Largest EVI2 amplitude cycle Phenology Timing Metrics**					
OGI	Onset Greenness Increase (Date of 15% greenness increase)	Day of year (January 1 of target year = 1)	1	−181–548	32767
50PCGI	50 Percent Greenness Increase (Date of 50% greenness increase)	Day of year (January 1 of target year = 1)	1	−181–548	32767
OGMx	Onset Greenness Maximum (Date of 90% greenness increase)	Day of year (January 1 of target year = 1)	1	−181–548	32767
Peak	Date of Cycle Peak	Day of year (January 1 of target year = 1)	1	1–366	32767
OGD	Onset Greenness Decrease (Date of 10% greenness decrease)	Day of year (January 1 of target year = 1)	1	−181–548	32767
50PCGD	50 Percent Greenness Decrease (Date of 50% greenness decrease)	Day of year (January 1 of target year = 1)	1	−181–548	32767
OGMn	Onset Greenness Minimum (Date of 85% greenness decrease)	Day of year (January 1 of target year = 1)	1	−181–548	32767
**Vegetation Indices**					
EVImax	Maximum EVI2 during vegetation cycle	—	0.0001	0–10000	32767
EVIamp	EVI2 Amplitude during vegetation cycle	—	0.0001	0–10000	32767
EVIarea	Integrated EVI2 during vegetation cycle	—	0.01	0–32766	32767
**Second Vegetation Cycle: Second Largest EVI2 amplitude cycle Phenology Timing Metrics**					
OGI_2	Onset Greenness Increase (Date of 15% greenness increase)	Day of year (January 1 of target year = 1)	1	−181–548	32767
50PCGI_2	50 Percent Greenness Increase (Date of 50% greenness increase)	Day of year (January 1 of target year = 1)	1	−181–548	32767
OGMx_2	Onset Greenness Maximum (Date of 90% greenness increase)	Day of year (January 1 of target year = 1)	1	−181–548	32767
Peak_2	Date of Cycle Peak	Day of year (January 1 of target year = 1)	1	1–366	32767
OGD_2	Onset Greenness Decrease (Date of 10% greenness decrease)	Day of year (January 1 of target year = 1)	1	−181–548	32767
50PCGD_2	50 Percent Greenness Decrease (Date of 50% greenness decrease)	Day of year (January 1 of target year = 1)	1	−181–548	32767
OGMn_2	Onset Greenness Minimum (Date of 85% greenness decrease)	Day of year (January 1 of target year = 1)	1	−181–548	32767
**Vegetation Indices**					
EVImax_2	EVI2 maximum during vegetation cycle	—	0.0001	0–10000	32767
EVIamp_2	EVI2 Amplitude during vegetation cycle	—	0.0001	0–10000	32767
EVIarea_2	EVI2 area during vegetation cycle	—	0.01	0–32766	32767
**Quality Assurance (QA)**					
QA	Quality Assurance for first vegetation cycle	—	1	1–4	—
QA_2	Quality Assurance for second vegetation cycle	—	1	1–4	—
numObs	Number of days with clear observations in calendar year	Days	1	0–366	32767

The structure of the dataset is as follows:

<Site Code__Site Full Name>

                            └─── PSLP_<Year>.nc

Here, “Site Code” and “Site Full Name” are the AmeriFlux site code and full site name, respectively, as presented in Table 1, and “Year” is the year of the estimated LSP metric from 2017 to 2021. Each netCDF file includes all 24 data layers for each year. Note that even though the PlanetScope imagery we use to create this dataset starts in 2016, we exclude this year from the PLSP dataset because the density of EVI2 times series is too sparse to estimate phenometrics with high confidence. To facilitate access to the location and boundary of each site, we include the GeoJSON file for each site as ancillary data.

## Technical Validation

A detailed technical validation of LSP metrics from PlanetScope imagery is presented in Moon *et al*.^26^. Results from that study demonstrated that LSP metrics estimated from PlanetScope image times series show strong agreement with LSP records derived from both HLS and PhenoCams, and that LSP metrics from PlanetScope capture information related to fine-scale variation in LSP that is not captured in medium or coarse spatial resolution LSP datasets^26^. Below we present additional validation that extends these previous results for the sites included in the PLSP dataset. First, we present images showing the mid-greenup date (50PCGI) and the EVI2 seasonal amplitude for three representative sites included in the dataset. Second, we present results showing the timing of peak EVI2 and associated PhenoCam images at the Jornada LTER site (US-xJR), which does not exhibit a clear annual cycle of vegetation activity. Third, we present phenological cycles at the Bouldin Island Alfalfa site (US-Bi1) to illustrate how our algorithm handles an extreme case with multiple growth cycles within a year. Fourth, we compare mid-greenup and mid-greendown dates (i.e., 50PCGI and 50PCGD, respectively) from PlanetScope imagery against an independent LSP dataset derived from HLS time series. Specifically, we compare the PLSP dataset against V011 of the MSLSP30NA^31^ data product (hereafter, MSLSP), which is publicly available via NASA’s Land Processes Distributed Active Archive Centre. Fifth, we compare 50PCGI and 50PCGD from the PLSP dataset against corresponding values estimated from PhenoCam *G*_*CC*_ time series.

**Fig. 2:**
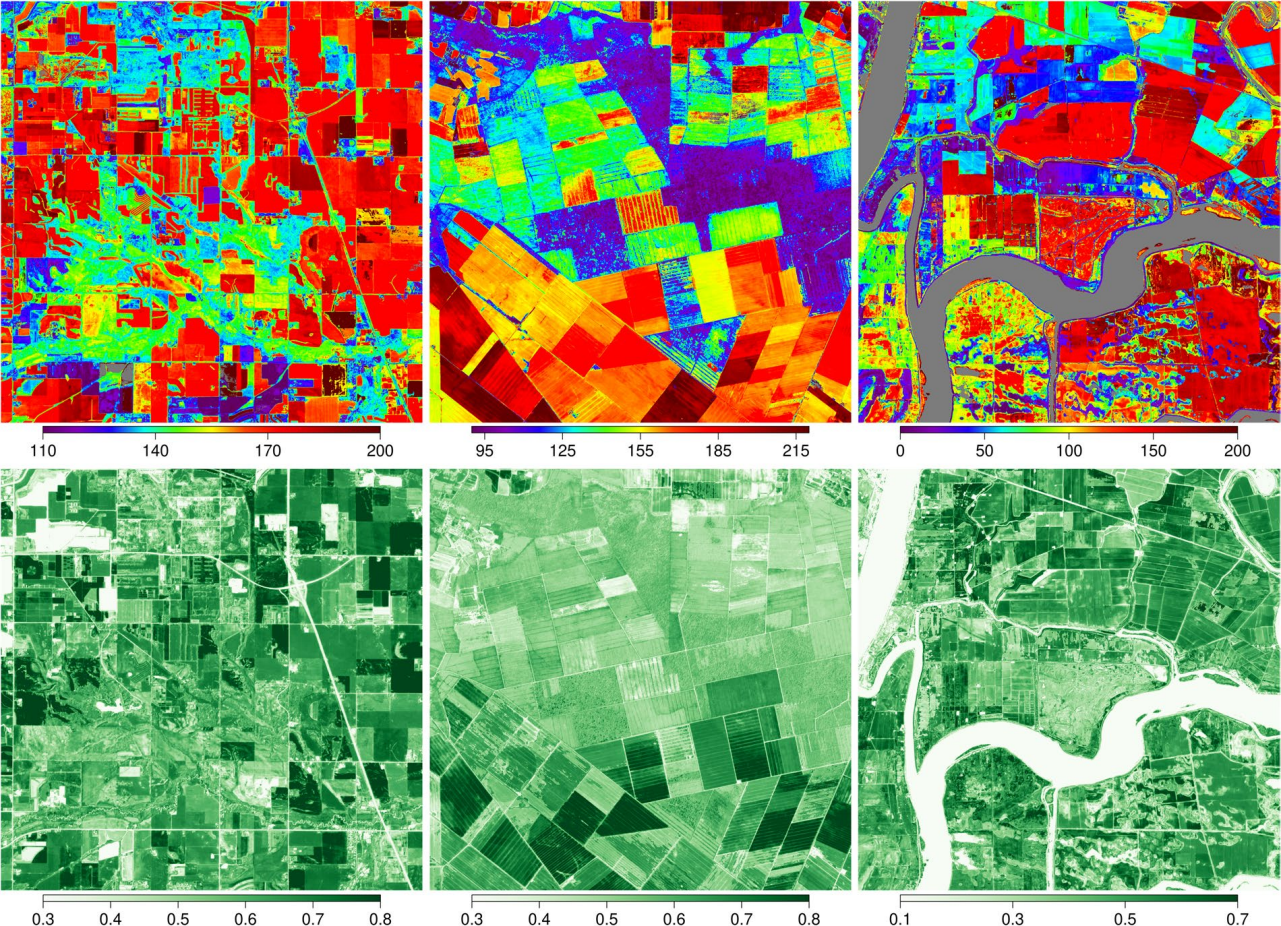
Greenup dates and EVI2 seasonal amplitudes in 2019. Each column represents a different site. Left: US-Ro5 (cropland); middle: US-NC2 (pine plantation); and right: US-Tw5 (wetland). The upper row shows the day of year corresponding to 50PCGI and the lower row shows the EVI2 amplitude at each 3 m pixel. The spatial extent for each image is 10 by 10 km.

Figure 2 shows representative images showing PLSP greenup dates and the seasonal amplitude of EVI2 for three sites with different landscapes included in the dataset. Each site has a land cover type (assigned by AmeriFlux) that is representative of the flux tower footprint. However, as is evident in these images, the 10 × 10 km^2^ areas surrounding these sites include a range of vegetation and land cover, which are manifested in the LSP data. For example, the primary land cover type for the US-Ro5 site (first column in Fig. 2) is cropland and greenup dates for most of the area surrounding the tower at the centre of the image occur around DOY 170. However, non-cropland areas (particularly in riparian areas) show greenup dates that are several weeks earlier, which reflects the impact of land management on vegetation phenology at the site. Further, the high spatial resolution of PlanetScope imagery captures fine-scale spatial variation in land surface phenology related to fine-scale variation in land cover (i.e., vegetated versus non-vegetated areas) associated with buildings, roads, and small water bodies. At the US-NC2 site (second column in Fig. 2), pine plantations (green and purple in the top row) show earlier greenup dates with low seasonal EVI2 amplitudes, while croplands show later greenup dates with large EVI2 amplitudes. The third column in Fig. 2 shows LSP images centred over AmeriFlux site US-Tw5, which is located in a wetland and is surrounded by heterogeneous land cover that includes a mix of wetlands, croplands, natural vegetation, and water bodies. Again, the LSP metrics in the PLSP dataset capture fine-scale variation in LSP across the site.

**Fig. 3:**
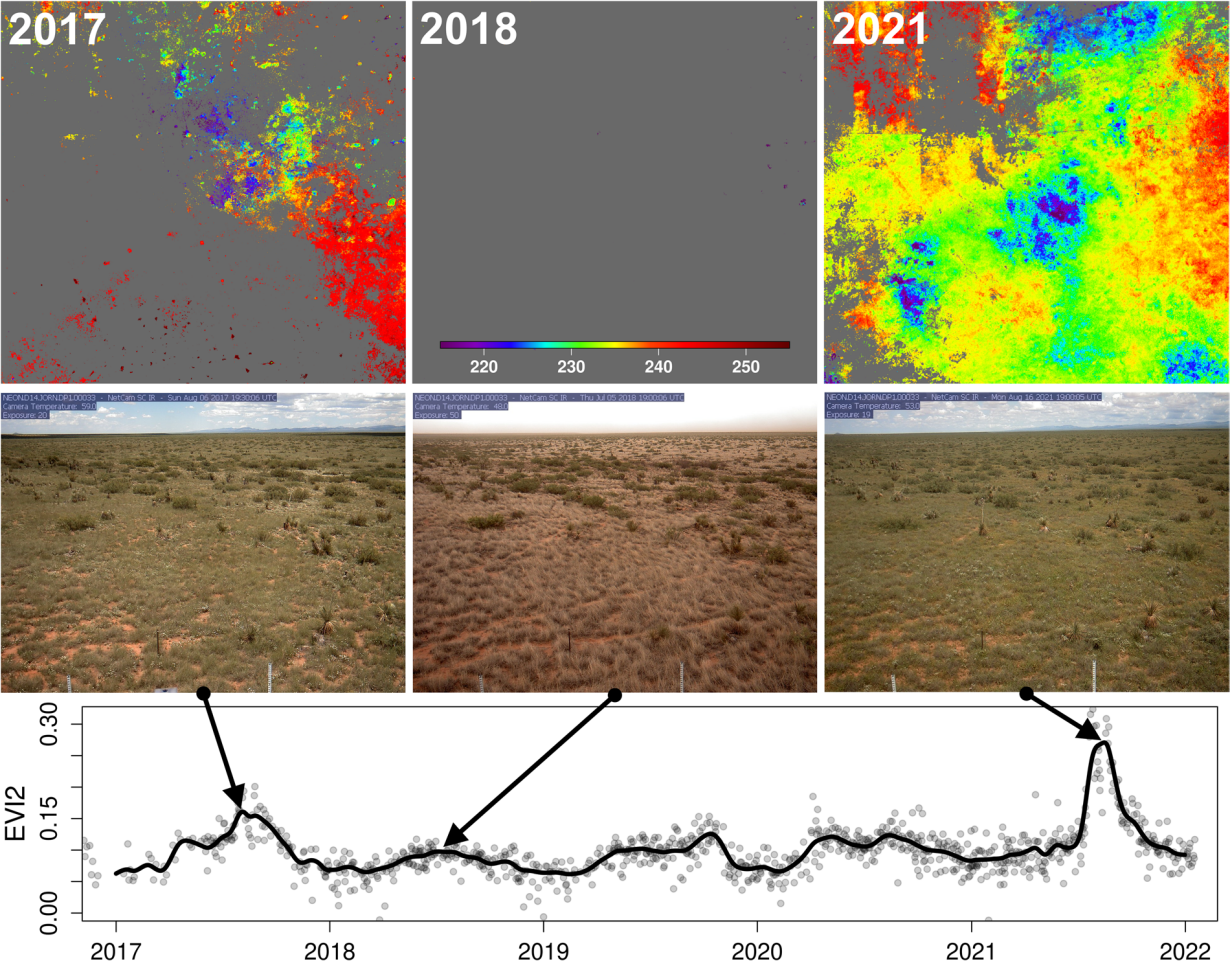
Five years of daily variation in vegetation activity at the Jornada LTER site (US-xJR). Images in the upper row show the day of year corresponding to the timing of maximum EVI2 in each PlanetScope pixel in 2017, 2018, and 2021. The PhenoCam is located at the centre of the site. The second row shows PhenoCam images acquired on dates corresponding to the maximum EVI2. The bottom panel shows EVI2 time series from a single PlanetScope pixel centred over the PhenoCam (dots: raw data; line: smoothing spline fit to data). Note, grey areas in the top row correspond to pixels where the seasonal amplitude of PlanetScope imagery did not exceed 0.1, which was the case for nearly all pixels in 2018.

Figure 3 presents PLSP images showing the timing maximum EVI2 at the Jornada LTER site in 2017, 2018, and 2021 (first row), PhenoCam images on dates corresponding to maximum EVI2 from PlanetScope (second row), and EVI2 time series from PlanetScope for 2017–2021 (third row). Phenology at this site shows either weak seasonality or no clear annual cycle of vegetation activity over the five-year period. In 2017, only 15% of pixels in the 10 by 10 km site exhibited sufficient phenological variation to be detected by the PLSP algorithm (i.e., 15% of pixels exhibited seasonal EVI2 amplitudes that exceeded 0.1). In 2018, less than 1% of pixels met this criterion. In contrast, over 75% of pixels in the site exhibited a measurable phenological cycle in 2021. This pattern is corroborated by PhenoCam images corresponding to the date of maximum PlanetScope EVI2 that are shown in the second row of Fig. 3, and demonstrates how the PLSP dataset is able to capture relatively subtle year-to-year variation in phenology.

**Fig. 4:**
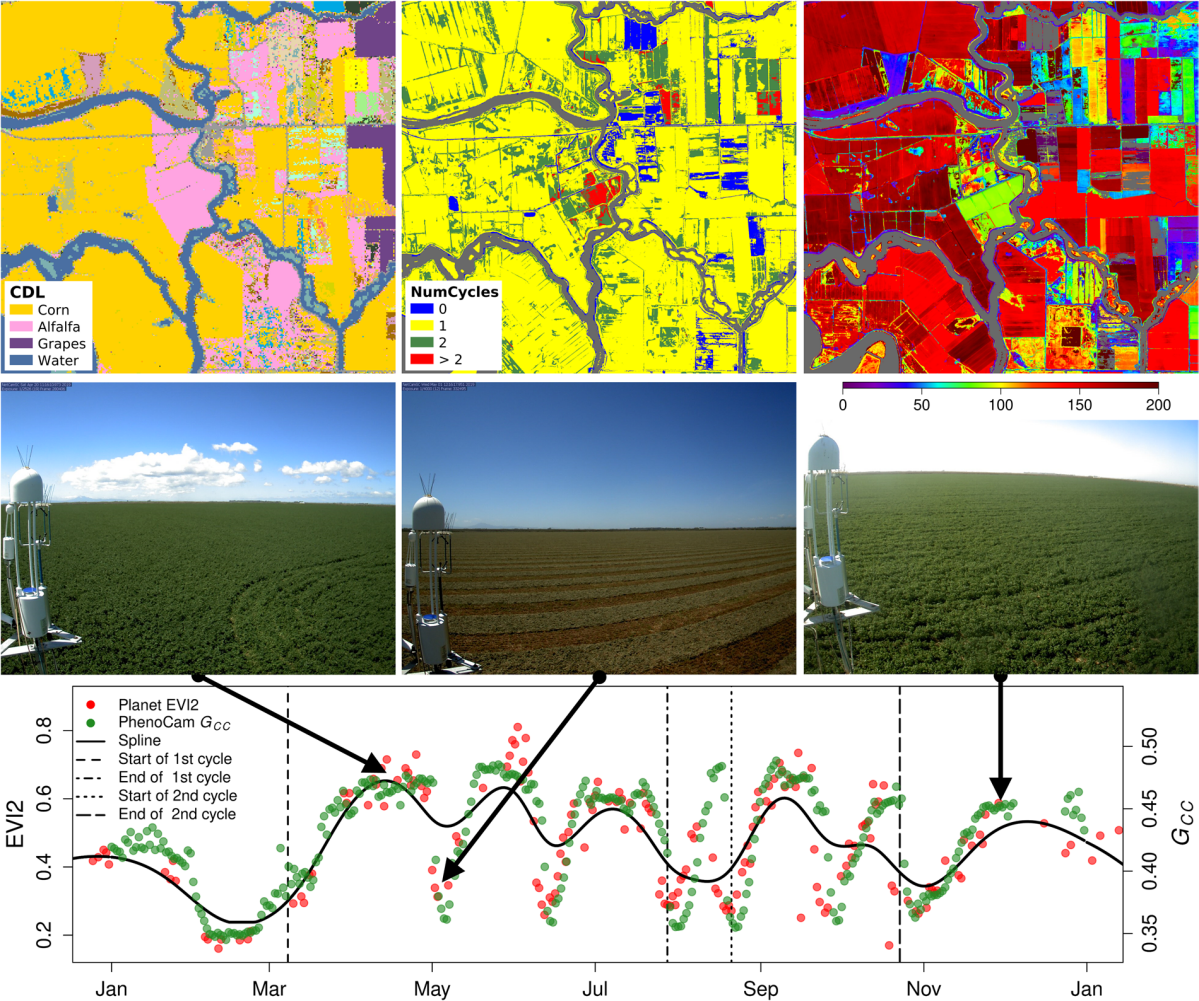
Phenological cycles at the Bouldin Island (Alfalfa) site (US-Bi1) in 2019. Images in the upper row show the Cropland Data Layer^36^, the number of phenological cycles, and the day of year corresponding to 50PCGI in 2019. The PhenoCam is located at the centre of the site. The second row shows PhenoCam images acquired on dates corresponding to the points identified by arrows in the bottom panel. The bottom panel shows EVI2 and *G*_*CC*_ time series from a single PlanetScope pixel centred over the PhenoCam and PhenoCam imagery.

Figure 4 shows PLSP and PhenoCam data from the US-Bi1 site, which is dominated by croplands and includes a variety of crop species. The PhenoCam’s field of view faces a field with alfalfa (second row), a crop with short growth cycles (generally less than two months) that is harvested multiple times per year. Corn fields occupy most of the landscape, exhibit relatively uniform phenology with one growth cycle, and have 50PCGI dates that range from DOY 150 to 190 (first row). PlanetScope EVI2 and PhenoCam *G*_*CC*_ time series both capture the short growth cycles of alfalfa (i.e., six growth cycles in 2019; third row). However, due to heuristics encoded in our algorithm (among others, that the start of each growth cycle is restricted to occur at least 30 days prior to the peak), the algorithm used to create the PLSP dataset does not provide a realistic representation of phenology for the alfalfa fields at this site (i.e., most pixels assigned as alfalfa are estimated to have three growth cycles, and the dataset provides phenometrics for only two growth cycles). That said, PlanetScope EVI2 time series clearly capture phenological dynamics in the field that are consistent with ground-based PhenoCam *G*_*CC*_ data. Hence, it should be possible to adjust our algorithm in future versions of the dataset to account for locations with more than two growing seasons each year.

**Fig. 5:**
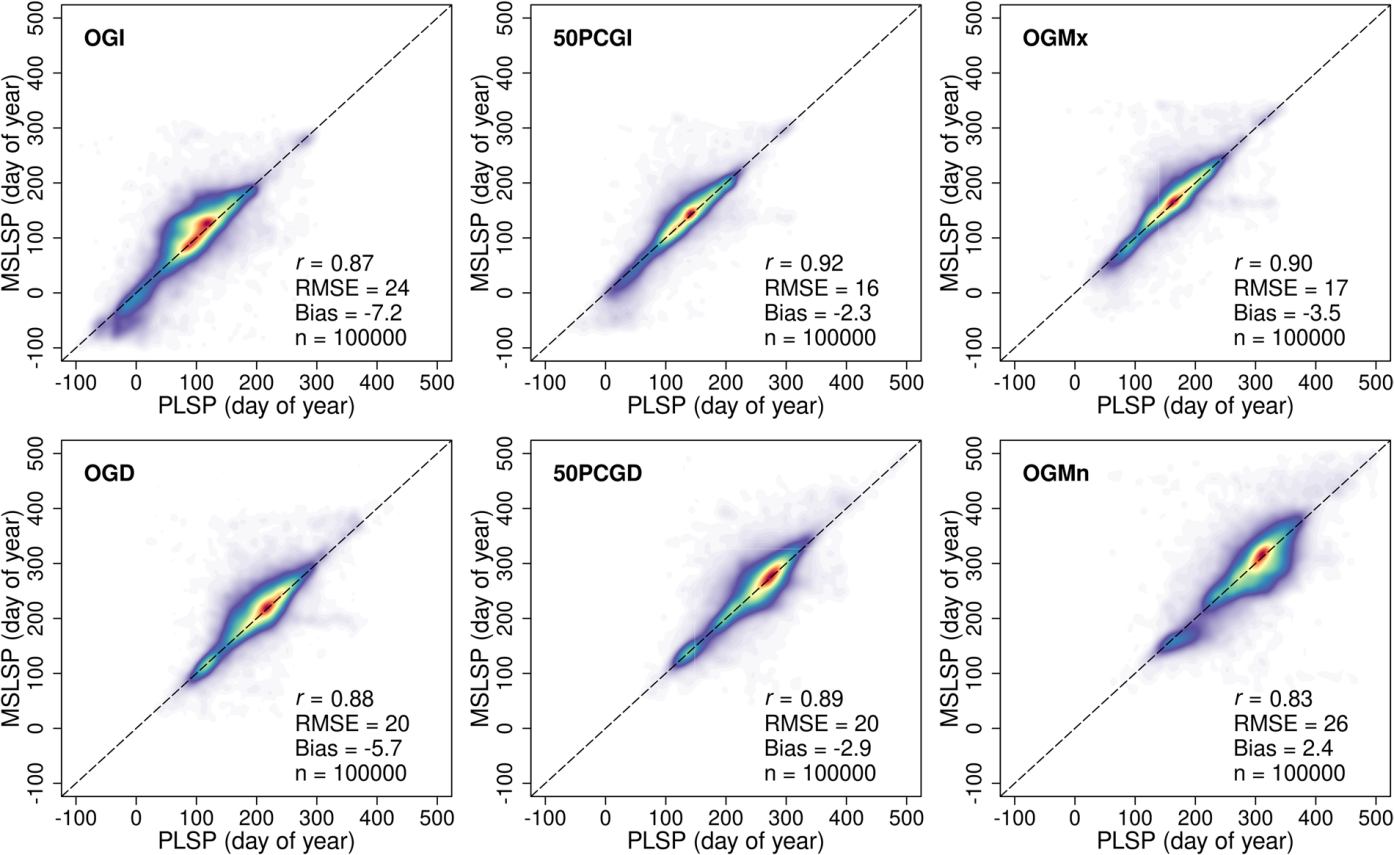
Comparison of PLSP and MSLSP metrics. Each plot compares average values for randomly selected 3 × 3 pixel windows in the MSLSP dataset against average values for co-located 30 × 30 pixel windows from the PLSP dataset (i.e., 90 by 90 m windows). Acronyms for the various LSP metrics are given in Table 2.

Figure 5 provides a comprehensive comparison of DOY phenometrics estimated from the MSLSP and PLSP datasets that includes data from 101 sites and six day-of-year phenometrics. Three sites were excluded (in Hawaii and Puerto Rico) because they were outside of the geographic coverage of the MSLSP dataset. This comparison uses average values from co-located 3 × 3 pixel windows from MSLSP images and 30 × 30 pixel windows from PLSP images (i.e., covering 90 by 90 m windows for both datasets) for 100,000 randomly sampled points across all sites from 2017 to 2019. Overall, there was strong agreement between LSP metrics from the PLPS and MSLSP datasets: the minimum correlation (*r*) is 0.83; the maximum root-mean-square error (RMSE) is 26 days; and the maximum bias (i.e., PLSP - MSLSP) is −7.2 days. Agreement was strongest for LSP metrics corresponding to the timing of maximum greenness and 50% greenup and greendown (i.e., OGMx, 50PCGI and 50PCGD, respectively), and was weakest for LSP metrics representing the start and end of growing seasons (i.e., OGI and OGMn), which showed modestly lower agreement and higher RMSEs. These lower agreements for OGI and OGMn can be attributed to the fact that these metrics are most susceptible to artefacts from image processing such as screening for snow- and cloud-contaminated pixels and determination of background EVI2 values.

**Fig. 6:**
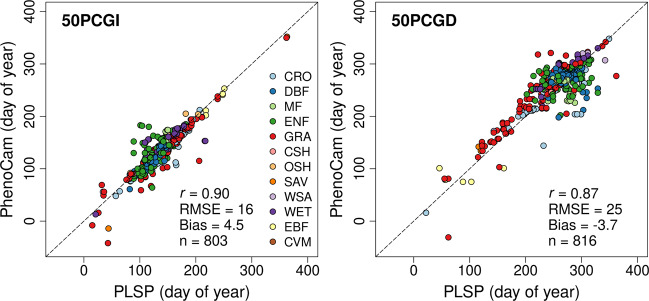
Comparison between PLSP and PhenoCam phenometrics. The comparison is based on average values for 5 × 5 pixel windows from PLSP images centred over each PhenoCam (i.e., 15 by 15 m). Colors indicate the IGBP land cover class assigned to each site by AmeriFlux or NEON.

As a final basis for assessing the PLSLP dataset, we compared phenometrics from PlanetScope to corresponding phenometrics from PhenoCam *G*_*CC*_ time series (Fig. 6). Across 5 years of PLSP measurements and 101 sites, the comparison includes PhenoCam imagery from 207 individual cameras (Supplementary Table 1), yielding 803 and 816 site-years of greenup and geendown dates, respectively. To perform this comparison, we used the average value of PLSP metrics for 5 × 5 pixel windows located 30 m north of each PhenoCam camera. We used this strategy because over 75% of the PhenoCams we used are oriented to face north. (Supplementary Table 1). And, given their proximity, the same PLSP value was used for comparisons of phenometrics from multiple ROIs within a single camera scene. Consistent with results comparing LSP metrics from the PLSP dataset against LSP metrics from the MSLSP dataset (i.e., Fig. 5), there is strong agreement between PLSP data and phenometrics from PhenoCam (*r* ≥ 0.87; RMSE ≤ 25 days; absolute bias ≤ 4.5 days). Note that even though phenometrics from PhenoCam and both PLSP and MSLSP are derived from different vegetation indices (i.e., *G*_*CC*_ versus EVI2), previous studies have demonstrated that phenological metrics derived from these two data sources show strong agreement^19,26^. Hence, once again, these results support the conclusion that LSP metrics included in the PLSLP dataset accurately capture the seasonality of vegetation activity at high spatial resolution across the sites included in the dataset.

## Usage Notes

At the time we processed the data (i.e., early 2022), time series of PlanetScope imagery did not cover the first six months of 2022. Hence, time series of EVI2 data did not cover the full period required by our algorithm to retrieve phenometrics in 2021. To overcome this, we used EVI2 time series data from 2021 in place of data that had not yet been acquired (i.e., we filled data from February to June in 2022 using data from 2021). To evaluate the impact of this strategy, we performed a sensitivity analysis (results not shown), which indicated that this approach had minimal impact on the quality of phenometrics in 2021. Also, unfortunately, our license agreement prevents us from distributing the quality-controlled and gap-filled EVI2 time series that we generated as intermediate products, which might also be useful to the community. As a compromise, all the source code that we used to generate these data are publicly available on our GitHub repository (see below), which allows interested readers to generate these data themselves.

## Supplementary information

PhenoCam sites info

## Data Availability

Python and R source code to download and process the PlanetScope imagery and generate the product can be obtained through a public repository at https://github.com/BU-LCSC/PLSP. R source code for generating the figures in the Technical Validation section is also available on the same repository.

## References

[1] Novick KA (2018). The AmeriFlux network: A coalition of the willing. Agr. Forest Meteoro..

[2] Pastorello G (2020). The FLUXNET2015 dataset and the ONEFlux processing pipeline for eddy covariance data. Sci Data.

[3] van Gorsel E (2018). Preface: OzFlux: a network for the study of ecosystem carbon and water dynamics across Australia and New Zealand. Biogeosciences.

[4] Baldocchi D (2001). FLUXNET: A New Tool to Study the Temporal and Spatial Variability of Ecosystem-Scale Carbon Dioxide, Water Vapor, and Energy Flux Densities. Bull. Amer. Meteor. Soc..

[5] Seyednasrollah B (2019). Tracking vegetation phenology across diverse biomes using Version 2.0 of the PhenoCam Dataset. Scientific Data.

[6] Richardson AD (2018). Tracking vegetation phenology across diverse North American biomes using PhenoCam imagery. Scientific Data.

[7] Piao S (2019). Plant phenology and global climate change: current progresses and challenges. Global Change Biology.

[8] Peano D (2021). Plant phenology evaluation of CRESCENDO land surface models – Part 1: Start and end of the growing season. Biogeosciences.

[9] Xu X, Riley WJ, Koven CD, Jia G, Zhang X (2020). Earlier leaf-out warms air in the north. Nat. Clim. Chang..

[10] Moon M, Li D, Liao W, Rigden AJ, Friedl MA (2020). Modification of surface energy balance during springtime: The relative importance of biophysical and meteorological changes. Agricultural and Forest Meteorology.

[11] Reed BC (1994). Measuring phenological variability from satellite imagery. Journal of Vegetation Science.

[12] White MA (2009). Intercomparison, interpretation, and assessment of spring phenology in North America estimated from remote sensing for 1982–2006. Global Change Biology.

[13] Zhang X (2003). Monitoring vegetation phenology using MODIS. Remote Sensing of Environment.

[14] Jonsson P, Eklundh L (2002). Seasonality extraction by function fitting to time-series of satellite sensor data. IEEE Transactions on Geoscience and Remote Sensing.

[15] Mahadevan, P. *et al*. A satellite-based biosphere parameterization for net ecosystem CO2 exchange: Vegetation Photosynthesis and Respiration Model (VPRM). *Global Biogeochemical Cycles* **22** (2008).

[16] Verma M (2014). Remote sensing of annual terrestrial gross primary productivity from MODIS: an assessment using the FLUXNET La Thuile data set. Biogeosciences.

[17] Verma M (2015). Improving the performance of remote sensing models for capturing intra- and inter-annual variations in daily GPP: An analysis using global FLUXNET tower data. Agricultural and Forest Meteorology.

[18] Xiao X (2004). Modeling gross primary production of temperate deciduous broadleaf forest using satellite images and climate data. Remote Sensing of Environment.

[19] Bolton DK (2020). Continental-scale land surface phenology from harmonized Landsat 8 and Sentinel-2 imagery. Remote Sensing of Environment.

[20] Bonan GB (2008). Forests and Climate Change: Forcings, Feedbacks, and the Climate Benefits of Forests. Science.

[21] Young AM (2021). Seasonality in aerodynamic resistance across a range of North American ecosystems. Agricultural and Forest Meteorology.

[22] Chu H (2021). Representativeness of Eddy-Covariance flux footprints for areas surrounding AmeriFlux sites. Agricultural and Forest Meteorology.

[23] Cheng Y (2020). Phenology of short vegetation cycles in a Kenyan rangeland from PlanetScope and Sentinel-2. Remote Sensing of Environment.

[24] Dixon DJ, Callow JN, Duncan JMA, Setterfield SA, Pauli N (2021). Satellite prediction of forest flowering phenology. Remote Sensing of Environment.

[25] Wang J (2020). Multi-scale integration of satellite remote sensing improves characterization of dry-season green-up in an Amazon tropical evergreen forest. Remote Sensing of Environment.

[26] Moon M, Richardson AD, Friedl MA (2021). Multiscale assessment of land surface phenology from harmonized Landsat 8 and Sentinel-2, PlanetScope, and PhenoCam imagery. Remote Sensing of Environment.

[27] Planet. Satellite Imagery and Archive. *Planet* https://planet.com/products/planet-imagery/ (2021).

[28] Dash J, Ogutu BO (2016). Recent advances in space-borne optical remote sensing systems for monitoring global terrestrial ecosystems. Progress in Physical Geography: Earth and Environment.

[29] Houborg R, McCabe MF (2018). A Cubesat enabled Spatio-Temporal Enhancement Method (CESTEM) utilizing Planet, Landsat and MODIS data. Remote Sensing of Environment.

[30] Jiang Z, Huete AR, Didan K, Miura T (2008). Development of a two-band enhanced vegetation index without a blue band. Remote Sensing of Environment.

[31] Friedl MA (2021). NASA EOSDIS Land Processes DAAC.

[32] Zhang X (2018). Generation and evaluation of the VIIRS land surface phenology product. Remote Sensing of Environment.

[33] Klosterman ST (2014). Evaluating remote sensing of deciduous forest phenology at multiple spatial scales using PhenoCam imagery. Biogeosciences.

[34] Moon M, Richardson AD, Milliman T, Friedl MA (2022). ORNL Distributed Active Archive Center.

[35] Sulla-Menashe D, Gray JM, Abercrombie SP, Friedl MA (2019). Hierarchical mapping of annual global land cover 2001 to present: The MODIS Collection 6 Land Cover product. Remote Sensing of Environment.

[36] USDA. USDA National Agricultural Statistics Service Cropland Data Layer. *crop-specific data layer* https://nassgeodata.gmu.edu/CropScape/ (2022).

