# Supplementary material for: A high spatial resolution land surface phenology dataset for AmeriFlux and NEON sites
Source: Sci Data. 2022 Jul 27;9:448. doi: 10.1038/s41597-022-01570-5 (PMC9329431; doi:10.1038/s41597-022-01570-5)
Supplement: Supplementary file 1 — PhenoCam sites info [file 41597_2022_1570_MOESM1_ESM.pdf]

Supplementary Information

Title

A high spatial resolution land surface phenology dataset for AmeriFlux and NEON sites

Authors

Minkyu Moon[1], Andrew D. Richardson[2,3], Thomas Milliman[4], Mark A. Friedl[1]

Affiliations

1. Department of Earth and Environment, Boston University, USA
2. School of Informatics, Computing & Cyber Systems, Northern Arizona University, USA
3. Center for Ecosystem Science and Society, Northern Arizona University, USA
4. Earth Systems Research Center, University of New Hampshire, USA

Table of contents

1. Supplementary Table 1

Supplementary Table 1. Site characteristics of the PhenoCam sites used in the present study. Note that PhenoCam cameras that are listed together with each of the AmeriFlux sites are the cameras that are located within the extent of the associated AmeriFlux site (i.e., within 10 km by 10 km boundary for each flux tower), including the cameras installed on the tower itself. Primary vegetation types are as follows: AG = agriculture; DB = deciduous broadleaf; EB = evergreen broadleaf; EN = evergreen needleleaf; GR = grassland; MX = mixed vegetation; SH = shrubs; TN = tundra; WL = wetland.

AmeriFlux Code	Camera name	Full site name	Latitude (°)	Longitude (°)	Primary Veg.	Camera Orient.	Acknowledgements
PR-xGU	NEON.D04.GUAN.DP1.00033	NEON Site - D04 (Atlantic Neotropical) Guanica Forest, Puerto Rico - tower top	17.9696	-66.8687	EB	N	The NEON Data Usage and Citation Policy can be found at: http://data.neonscience.org/data-policy. NEON is a project sponsored by the National Science Foundation and operated under cooperative agreement by Battelle.
	NEON.D04.GUAN.DP1.00042	NEON Site - D04 (Atlantic Neotropical) Guanica Forest, Puerto Rico - mid-tower	17.9696	-66.8687	EB	N	The NEON Data Usage and Citation Policy can be found at: http://data.neonscience.org/data-policy. NEON is a project sponsored by the National Science Foundation and operated under cooperative agreement by Battelle.
PR-xLA	NEON.D04.LAJA.DP1.00033	NEON Site - D04 (Atlantic Neotropical) Lajas Experimental Station, Puerto Rico - tower top	18.0213	-67.0769	EB	N	The NEON Data Usage and Citation Policy can be found at: http://data.neonscience.org/data-policy. NEON is a project sponsored by the National Science Foundation and operated under cooperative agreement by Battelle.
	NEON.D04.LAJA.DP1.00042	NEON Site - D04 (Atlantic Neotropical) Lajas Experimental Station, Puerto Rico - mid-tower	18.0213	-67.0769	EB	N	The NEON Data Usage and Citation Policy can be found at: http://data.neonscience.org/data-policy. NEON is a project sponsored by the National Science Foundation and operated under cooperative agreement by Battelle.
US-ARM	armoklahoma	ARM Climate Research Facility, Billings, Oklahoma	36.6058	-97.4888	AG		
	southerngreatplains	ARM Southern Great Plains Central Facility, Billings, Oklahoma, USA	36.6058	-97.4888	AG	N	Research at the site is supported by the Office of Biological and Environmental Research of the US Department of Energy under contract No. DE-AC02-05CH11231 as part of the Atmospheric Radiation Measurement Program (ARM),
US-Bi1/ US-Bi2	bouldinalfalfa	Alfalfa on peat soils in the SSJ river delta CA	38.0985	-121.4993	AG	NW	California Department of Fish and Wildlife
	bouldincorn	Corn on peat SSJ River Delta Bouldin Island CA	38.1090	-121.5350	AG	WNW	California Department of Fish and Wildlife
	statenrice1	Rice field eddy covariance tower at Staten Island, CA	38.1235	-121.5490	AG	W	
US-BMM	bozeman	Bangtail Study Area, Montana State University, Montana	45.7831	-110.7778	GR	N	Research at the Bozeman site is supported by Colorado State University and the AmericaView program (grants G13AC00393, G11AC20461, G15AC00056) with phenocam equipment and deployment sponsored by the Department of Interior North Central Climate Science Center.
US-CF1/ US-CF2/ US-CF3/ US-CF4	cafboydnorthltar01	CAF-LTAR Boyd North; LTAR tower at Boyd North, R. J. Cook Agronomy Farm, Pullman, Washington, USA	46.7551	-117.1261	AG		The USDA-ARS supported Cook Agronomy Farm Long-Term Agro-ecosystem Research site provided data for this work.
	cafboydsouthltar01	CAF-LTAR Boyd South; LTAR tower at Boyd South, R. J. Cook Agronomy Farm, Pullman, Washington, USA	46.7518	-117.1285	AG	SSW	The USDA-ARS supported Cook Agronomy Farm Long-Term Agro-ecosystem Research site provided data for this work.
	cafcookeastltar01	CAF-LTAR, Business as usual site, Cook East; LTAR tower at Cook East, R. J. Cook Agronomy Farm, Pullman, Washington, USA	46.7815	-117.0821	AG	SSW	The USDA-ARS supported Cook Agronomy Farm Long-Term Agro-ecosystem Research site provided data for this work.
	cafcookwestltar01	CAF-LTAR Aspirational, Cook West; LTAR tower at Cook West, R. J. Cook Agronomy Farm, Pullman, Washington, USA	46.7840	-117.0908	AG	SSE	The USDA-ARS supported Cook Agronomy Farm Long-Term Agro-ecosystem Research site provided data for this work.
US-Ha1/ US-Ha2/ US-xHA	bbc1	Hardwood Walk-up Tower, Harvard Forest, Petersham, Massachusetts	42.5351	-72.1744	DB	NE	
	bbc2	LPH Tower, Harvard Forest, Petersham, Massachusetts	42.5420	-72.1850	DB	WNW	
	harvard	EMS Tower, Harvard Forest, Petersham, Massachusetts	42.5378	-72.1715	DB	N	The Harvard EMS site is supported is an AmeriFlux core site supported by the AmeriFlux Management Project with funding by the U.S. Department of Energy's Office of Science under Contract No. DE-AC02-05CH11231, and a part of the Harvard Forest LTER site supported by the National Science Foundation (DEB-1237491).
	harvardbarn	Barn Tower, Camera 1, Harvard Forest, Petersham, Massachusetts	42.5353	-72.1899	EN	N	Research at Harvard Forest is partially supported through the National Science Foundation's LTER program (DEB-1237491).
	harvardbarn2	Barn Tower, Camera 2, Harvard Forest, Petersham, Massachusetts	42.5353	-72.1899	DB	N	Research at Harvard Forest is partially supported through the National Science Foundation's LTER program (DEB-1237491).
	harvardblo	Below-canopy camera, EMS Tower, Harvard Forest, Petersham, Massachusetts	42.5378	-72.1715	DB	NW	The Harvard EMS site is supported is an AmeriFlux core site supported by the AmeriFlux Management Project with funding by the U.S. Department of Energy's Office of Science under Contract No. DE-AC02-05CH11231, and a part of the Harvard Forest LTER site supported by the National Science Foundation (DEB-1237491).
US-Ha1/ US-Ha2/ US-xHA	harvardems2	EMS Tower, Harvard Forest, Petersham, Massachusetts - new camera	42.5378	-72.1715	DB	N	The Harvard EMS site is supported is an AmeriFlux core site supported by the AmeriFlux Management Project with funding by the U.S. Department of Energy's Office of Science under Contract No. DE-AC02-05CH11231, and a part of the Harvard Forest LTER site supported by the National Science Foundation (DEB-1237491).
	harvardfarmnorth	Harvard Farm, Petersham, Massachusetts	42.5205	-72.1822	GR	NE	Research at Harvard Forest is partially supported through the National Science Foundation's LTER program (DEB-1237491).
	harvardfarmsouth	Harvard Farm, Petersham, Massachusetts	42.5225	-72.1823	GR	NE	Research at Harvard Forest is partially supported through the National Science Foundation's LTER program (DEB-1237491).

AmeriFlux Code	Camera name	Full site name	Latitude (°)	Longitude (°)	Primary Veg.	Camera Orient.	Acknowledgements
	harvardgarden	Common Garden, Harvard Forest, Petersham, MA	42.5291	-72.1863			
	harvardhemlock	Hemlock Tower, Harvard Forest, Petersham, Massachusetts	42.5390	-72.1800	EN	NE	Research at Harvard Forest is partially supported through the National Science Foundation's LTER program (DEB-1237491), and Dept. of Energy Office of Science (BER)
	harvardhemlock2	Hemlock Tower, Harvard Forest, Petersham, MA, USA	42.5394	-72.1780	EN	SW	Research at Harvard Forest is partially supported through the National Science Foundation's LTER program (DEB-1237491).
	harvardlph	LPH Tower, Harvard Forest, Petersham, Massachusetts	42.5420	-72.1850	DB	NW	Research at Harvard Forest is partially supported through the National Science Foundation's LTER program (DEB-1237491).
	NEON.D01.HARV.DP1.00033	NEON Site - D01 (Northeast) Harvard Forest, Massachusetts - tower top	42.5369	-72.1727	DB	N	The NEON Data Usage and Citation Policy can be found at: http://data.neonscience.org/data-policy. NEON is a project sponsored by the National Science Foundation and operated under cooperative agreement by Battelle.
	NEON.D01.HARV.DP1.00042	NEON Site - D01 (Northeast) Harvard Forest, Massachusetts - mid-tower	42.5369	-72.1727	MX	N	The NEON Data Usage and Citation Policy can be found at: http://data.neonscience.org/data-policy. NEON is a project sponsored by the National Science Foundation and operated under cooperative agreement by Battelle.
	witnesstree	Witness Tree, Barn Tower, Harvard Forest, Petersham, Massachusetts	42.5356	-72.1896	DB		
US-HB1/ US-HB2/ US-HB3	brackishimpoundment	Managed brackish waterfowl impoundment	33.3482	-79.2322	WL	NE	Clemson University College of Agriculture, Forestry and Life Sciences
	hobcawclearcutlongleaf	Hobcaw Longleaf Pine Clearcut	33.3486	-79.2320	EN	N	
	hobcawmaturelongleaf	Hobcaw Mature Longleaf Pine	33.3242	-79.2439	EN	NNE	
	northinletsaltmarsh	North Inlet-Winyah Bay Salt Marsh, Georgetown, SC	33.3455	-79.1957	WL	N	We thank the Belle W. Baruch Foundation for hosting the site and the North Inlet - Winyah Bay National Estuarine Research Reserve (NI-WB NERR) and the South Carolina Sea Grant Consortium (grant NA18OAR4170091) for support.
US-Ho1	howland1	Main Tower (Mature stand), Howland Forest, Howland, Maine	45.2041	-68.7403	EN	N	Research at Howland Forest is supported by the Office of Science (BER), US Department of Energy, and the USDA Forest Service's Northern Research Station.
	howland2	North Tower (Regrowing clearcut, ca. 1990), Howland Forest, Howland, Maine	45.2128	-68.7418	DB	N	Research at Howland Forest is supported by the Office of Science (BER), US Department of Energy, and the USDA Forest Service's Northern Research Station.
US-ICs	imcrkfen	Imnavait Creek Fen, AON IC_1523, Alaska	68.6058	-149.3110	TN	N	Research at the Imnavait Watershed is funded by the National Science Foundation Division of Polar Programs Arctic Observatory Network (grants 1107892 and 1446216).
	imcrkridge0	Imnavait Creek Ridge, camera-0, AON IC_1991, Alaska	68.6068	-149.2958	TN	SE	Research at the Imnavait Watershed is funded by the National Science Foundation Division of Polar Programs Arctic Observatory Network (grants 1107892 and 1446216).
	imcrkridge1	Imnavait Creek Ridge, camera-1, AON IC_1991, Alaska	68.6068	-149.2958	TN	N	Research at the Imnavait Watershed is funded by the National Science Foundation Division of Polar Programs Arctic Observatory Network (grants 1107892 and 1446216).
	imcrktussock	Imnavait Creek Tussock, AON IC_1993, Alaska	68.6063	-149.3041	TN	N	Research at the Imnavait Watershed is funded by the National Science Foundation Division of Polar Programs Arctic Observatory Network (grants 1107892 and 1446216).
US-KFS/ US-xUK	kansas	KU Field Station, University of Kansas, Kansas	39.0561	-95.1907	GR	NE	The US-KFS site is sponsored by the U.S. Department of Energy under a subcontract from DE-AC02-05CH11231.
	NEON.D06.UKFS.DP1.00033	NEON Site - D06 (Prairie Peninsula) The University of Kansas Field Station, Kansas - tower top	39.0404	-95.1922	DB	N	The NEON Data Usage and Citation Policy can be found at: http://data.neonscience.org/data-policy. NEON is a project sponsored by the National Science Foundation and operated under cooperative agreement by Battelle.
	NEON.D06.UKFS.DP1.00042	NEON Site - D06 (Prairie Peninsula) The University of Kansas Field Station, Kansas - mid-tower	39.0404	-95.1922	DB	N	The NEON Data Usage and Citation Policy can be found at: http://data.neonscience.org/data-policy. NEON is a project sponsored by the National Science Foundation and operated under cooperative agreement by Battelle.
US-Me2	oregonMP	Metolius intermediate pine/US-Me2, near Sisters, Oregon	44.4523	-121.5574	EN	NE	Support for US-Me2 is provided from the Metolius Core Site Cluster by the DOE Office of Science Ameriflux Network Management Project
	oregonmp1	Ponderosa pine bud, Metolius mature ponderosa pine/US-Me2, near Sisters, OR	44.4523	-121.5574	EN	N	Funding for this AmeriFlux core site was provided by the U.S. Department of Energy's Office of Science
US-Me6	oregonYP	Metolius New Young Pine/US-Me6, near Sisters, Oregon	44.3238	-121.6060	EN	N	Support for US-Me6 is provided from the Metolius Core Site Cluster by the DOE Office of Science Ameriflux Network Management Project
	oregonYPbud	ponderosa pine bud, Metolius New Young Pine/US-Me-6, near Sisters, OR	44.3238	-121.6060	EN	N	Funding for this AmeriFlux core site was provided by the U.S. Department of Energy's Office of Science
US-MMS	morganmonroe	Morgan Monroe State Forest, Indiana	39.3231	-86.4131	DB	N	Research at the Morgan-Monroe Ameriflux site is supported by the US Department of Energy, Office of Science, Office of Biological and Environmental Research through the Ameriflux Management Project administered by Lawrence Berkeley National Lab
	morganmonroe2	Morgan Monroe State Forest, Indiana	39.3231	-86.4131	DB	N	Research at the Morgan-Monroe Ameriflux site is supported by the US Department of Energy, Office of Science, Office of Biological and Environmental Research through the Ameriflux Management Project administered by Lawrence Berkeley National Lab
US-Mpj	usmpj	Heritage Land Conservancy, Pinyon-Juniper Site near Mountainair, NM	34.4385	-106.2544	SH	N	
US-Myb/ US-Sne/ US-Snf	mayberry	Mayberry Slough, Twitchell Island, Antioch, California	38.0498	-121.7651	WL	W	
	sherman	Twitchell Island, Antioch, California	38.0366	-121.7540	GR	W	
	shermanbarn	Pasture on Sherman Island in the Sacramento-San Joaquin River Delta, CA	38.0400	-121.7270	GR	WNW	California Department of Fish and Wildlife
	siwetland	Sherman Island	38.0369	-121.7546	WL	W	

AmeriFlux Code	Camera name	Full site name	Latitude (°)	Longitude (°)	Primary Veg.	Camera Orient.	Acknowledgements
US-NC2/ US-NC3	ncloblolly1994	Main tower, NC2 at Parker Tract, Plymouth, NC	35.8031	-76.6679	EN	NW	This site is supported by DOE BER-TES awards number 7090112 and 11-DE-SC-0006700, USDA NIFA Grant 2011-67009- 20089, US Forest Service Eastern Forest Environmental Threat Assessment Center Grant 08-JV-11330147-038, and the PINEMAP project. The Pine Integrated Network: Education, Mitigation, and Adaptation project (PINEMAP) is a Coordinated Agricultural Project funded by the USDA National Institute of Food and Agriculture, Award #2011-68002-30185.
US-NC4	alligatorriver	Alligator River National Wildlife Refuge, North Carolina	35.7879	-75.9038	DB	N	Research at the Alligator River flux site is supported by DOE NICCR (award 08-SC-NICCR-1072), DOE-TES (awards 11-DE-SC-0006700 and 7090112), USDA Forest Service (award 13-JV-11330110-081) and USDA-NIFA (award 2014-67003-22068).
US-Ne1/ US-Ne2/ US-Ne3	mead1	US-Ne1 Mead - irrigated continuous maize site	41.1651	-96.4766	AG		
	mead2	US-Ne2 Irrigated maize-soybean rotation	41.1649	-96.4701	AG		
	mead3	US-Ne3 Rainfed maize-soybean rotation	41.1797	-96.4397	AG		
	meadpasture	Platte River - High Plains Aquifer LTAR Pasture (BAU)	41.1447	-96.4616	AG		
	meadpasturese	Platte River - High Plains Aquifer LTAR Pasture (BAU), southeast view	41.1447	-96.4616	AG	SE	
	meadpasturesw	Platte River - High Plains Aquifer LTAR Pasture (BAU), southwest view	41.1447	-96.4616	AG	SW	
US-NR1/ US-xNW	NEON.D13.NIWO.DP1.00033	NEON Site - D13 (Southern Rockies and Colorado Plateau) Niwot Ridge Mountain Research Station, Colorado - tower top	40.0543	-105.5824	TN	N	The NEON Data Usage and Citation Policy can be found at: http://data.neonscience.org/data-policy. NEON is a project sponsored by the National Science Foundation and operated under cooperative agreement by Battelle.
	NEON.D13.NIWO.DP1.00042	NEON Site - D13 (Southern Rockies and Colorado Plateau) Niwot Ridge Mountain Research Station, Colorado - mid-tower	40.0543	-105.5824	TN	N	The NEON Data Usage and Citation Policy can be found at: http://data.neonscience.org/data-policy. NEON is a project sponsored by the National Science Foundation and operated under cooperative agreement by Battelle.
	niwot2	Niwot Ridge Mountain Research Station, Roosevelt National Forest, Colorado	40.0329	-105.5470	EN	N	The US-NR1 AmeriFlux site is currently supported by the U.S. DOE, Office of Science through the AmeriFlux Management Project (AMP) at Lawrence Berkeley National Laboratory under Award Number 7094866.
	niwot3	Niwot Ridge Mountain Research Station, Roosevelt National Forest, Colorado	40.0329	-105.5470	EN	N	The US-NR1 AmeriFlux site is currently supported by the U.S. DOE, Office of Science through the AmeriFlux Management Project (AMP) at Lawrence Berkeley National Laboratory under Award Number 7094866.
	niwot4	Niwot Ridge Mountain Research Station, Roosevelt National Forest, Colorado	40.0329	-105.5470	EN	E	The US-NR1 AmeriFlux site is currently supported by the U.S. DOE, Office of Science through the AmeriFlux Management Project (AMP) at Lawrence Berkeley National Laboratory under Award Number 7094866.
	niwot5	Niwot Ridge, Colorado, USA	40.0329	-105.5470	EN	SE	The US-NR1 AmeriFlux site is currently supported by the U.S. DOE, Office of Science through the AmeriFlux Management Project (AMP) at Lawrence Berkeley National Laboratory under Award Number 7094866.
	niwotridge	Niwot Ridge Mountain Research Station, Roosevelt National Forest, Colorado	40.0329	-105.5470	EN	N	The US-NR1 AmeriFlux site is currently supported by the U.S. DOE, Office of Science through the AmeriFlux Management Project (AMP) at Lawrence Berkeley National Laboratory under Award Number 7094866.
US-Rms	arsgreatbasinltar177	ARS, Great Basin LTAR, ARTRV/SYORU community, Reynolds Creek, Idaho	43.0645	-116.7484	SH	N	his research at the Reynolds Creek Experimental Watershed and the Great Basin Long-Term Agroecosystem Research (LTAR) site is funded by the USDA Agricultural Research Service (ARS) through ARS Project Numbers 2052-13610-011-00-D and 2052-13610-012-00-D and is a collaborative contribution to the LTAR Network. This camera location occurs on rangelands managed by the USDI Bureau of Land Management and is also co-located on a site within the Reynolds Creek Critical Zone Observatory which is funded by the National Science Foundation under Grant Number EAR-1331872.
US-Ro4/ US-Ro5/ US-Ro6	rosemount	Rosemount Agricultural Experiment Station, University of Minnesota, Rosemount, Minnesota	44.7143	-93.0898	AG	N	
	rosemountcons	Rosemount Conservation Agriculture Site Rosemount, MN 55068	44.6946	-93.0578	AG	N	Site acknowledgements: Long Term Agroecosystem Research (LTAR) Network, Ameriflux, USDA - ARS, University of Minnesota
US-Ro4/ US-Ro5/ US-Ro6	rosemountconv	Rosemount Conventional Agricultural Management Site Rosemount, MN 55068	44.6910	-93.0576	AG	N	Site acknowledgements: Long Term Agroecosystem Research (LTAR) Network, Ameriflux, USDA - ARS, University of Minnesota
	rosemountg21	G21, Rosemount Agricultural Experiment Station, University of Minnesota, Rosemount, Minnesota	44.7143	-93.0898	AG	N	The US-Ro1 AmeriFlux core site is currently supported by the US DOE, Office of Science through the AmeriFlux Management Project (AMP) at Lawrence Berkeley National Laboratory and base funding from USDA-ARS
	rosemountnprs	Native Prairie Restoration Site, Rosemount Agricultural Experiment Station, University of Minnesota, Rosemount, Minnesota	44.6781	-93.0723	AG	N	The US-Ro4 AmeriFlux core site is currently supported by the US DOE, Office of Science through the AmeriFlux Management Project (AMP) at Lawrence Berkeley National Laboratory and base funding from USDA-ARS
US-Ro5/ US-Ro6	rosemountc6	C6, Rosemount Agricultural Experiment Station, University of Minnesota, Rosemount, Minnesota	44.7288	-93.0888	AG	N	The US-Ro2 AmeriFlux core site is currently supported by the US DOE, Office of Science through the AmeriFlux Management Project (AMP) at Lawrence Berkeley National Laboratory and base funding from USDA-ARS
US-Rws	arsgreatbasinltar098	ARS, Great Basin LTAR, ARTRW8 community, Reynolds Creek, Idaho	43.1675	-116.7132	SH	N	This research at the Reynolds Creek Experimental Watershed and the Great Basin Long-Term Agroecosystem Research (LTAR) site is funded by the USDA Agricultural Research Service (ARS) through ARS Project Numbers 2052-13610-011-00-D and 2052-13610-012-00-D and is a collaborative contribution to the LTAR Network. This camera location occurs on rangelands managed by the USDI Bureau of Land Management and is also co-located on a site within the Reynolds Creek Critical Zone Observatory which is funded by the National Science Foundation under Grant Number EAR-1331872.
	arsgreatbasinltar117	ARS, Great Basin LTAR, ARAR8 community, Reynolds Creek, Idaho	43.1432	-116.7357	SH	N	This research at the Reynolds Creek Experimental Watershed and the Great Basin Long-Term Agroecosystem Research (LTAR) site is funded by the USDA Agricultural Research Service (ARS) through ARS Project Numbers 2052-13610-011-00-D and 2052-13610-012-00-D and is a collaborative contribution to the LTAR Network. This camera location occurs on rangelands managed by the USDI Bureau of Land Management and is also co-located on a site within the Reynolds Creek Critical Zone Observatory which is funded by the National Science Foundation under Grant Number EAR-1331872.

AmeriFlux Code	Camera name	Full site name	Latitude (°)	Longitude (°)	Primary Veg.	Camera Orient.	Acknowledgements
US-Seg	sevilletanewgrass	Unburned Grassland Site, Sevilleta Long Term Ecological Research Site, New Mexico	34.3580	-106.6799	GR	N	Ameriflux
US-Seg/ US-Ses	sevilletagrass	Grassland site, Sevilleta Long Term Ecological Research Site, New Mexico	34.3604	-106.7002	GR		Support is provided by the Sevilleta Field Station and the University of New Mexico
	sevilletashrub	Shrubland site, Sevilleta Long Term Ecological Research Site, New Mexico	34.3350	-106.7445	SH	N	Support is provided by the Sevilleta Field Station and the University of New Mexico
	sevMRME10L	Plot 10 Large Treatment, Monsoon Rainfall Manipulation Experiment, Sevilleta National Wildlife Refuge, New Mexico	34.3438	-106.7269	GR	N	Support is provided by the Sevilleta Field Station and the University of New Mexico
	sevMRME11C	Plot 11 Control Treatment, Monsoon Rainfall Manipulation Experiment, Sevilleta National Wildlife Refuge, New Mexico	34.3438	-106.7268	GR	N	Support is provided by the Sevilleta Field Station and the University of New Mexico
	sevMRME1S	Plot 1 Small Treatment, Monsoon Rainfall Manipulation Experiment, Sevilleta National Wildlife Refuge, New Mexico	34.3442	-106.7272	GR	N	Support is provided by the Sevilleta Field Station and the University of New Mexico
US-SRG/ US-SRM	srg	Santa Rita Experimental Range Grassland AmeriFlux site, southern Arizona	31.7894	-110.8276	GR		
	srm	Mesquite Savanna, Southern Arizona	31.8214	-110.8661		N	USDA-ARS and AmeriFlux core site funding from DOE
US-Syv	sylvania	Sylvania Wilderness, Wisconsin	46.2420	-89.3480	EN	N	Support for US-Syv is provided from the ChEAS Core Site Cluster by the DOE Office of Science Ameriflux Network Management Project
US-Ton/ US-Var	tonzi	Tonzi Ranch, Amador County, California	38.4309	-120.9659	DB	NW	Funding for AmeriFlux core site data was provided by the U.S. Department of Energy's Office of Science.
	vaira	Vaira Ranch, Amador County, California	38.4133	-120.9506	GR	NW	
US-Tw1/ US-Tw3/ US-Tw4/ US-Tw5	eastend	Twitchell Island, Antioch, California	38.1027	-121.6413	WL	W	
	eastend2	Twitchell Island, Antioch, California	38.1027	-121.6413	WL		
	twitchell	Twitchell Island, Antioch, California	38.1087	-121.6530	AG	W	
	twitchellalfalfa	Twitchell Island, Antioch, California, USA	38.1154	-121.6467	AG	W	
	twitchellalfalfa2	Twitchell Island, Antioch, California, USA	38.1154	-121.6467	AG		
	westpond	Twitchell Island, Antioch, California	38.1074	-121.6469	WL	W	
US-UMB/ US-UMd	umichbiological	University of Michigan Biological Station, near Pellston, Michigan	45.5598	-84.7138	DB	N	Primary support for the University of Michigan AmeriFlux Core Site (US-UMB) provided by the Department of Energy Office of Science. Infrastructure support provided by the University of Michigan Biological Station.
	umichbiological2	FASET Tower, University of Michigan Biological Station, near Pellston, Michigan	45.5625	-84.6976	DB	N	Primary support for the University of Michigan AmeriFlux Core Site (US-UMd) provided by the Department of Energy Office of Science. Infrastructure support provided by the University of Michigan Biological Station.
US-Vcm	vallesburnedconifer	Subalpine Burned Conifer Site, Valles Cauldera National Preserve, New Mexico	35.8884	-106.5321	UN		AmeriFlux
US-Vcp	vallesponderosapine	Ponderosa Pine Site, Valles Cauldera National Preserve, New Mexico	35.8640	-106.5964	EN		Ameriflux
US-Vcs	vallesmixedconifer	Subalpine Mixed Conifer Site, Valles Cauldera National Preserve, New Mexico	35.9192	-106.6142	EB		Ameriflux
US-WCr	willowcreek	Willow Creek, Chequamegon-Nicolet National Forest, Wisconsin	45.8060	-90.0791	DB	N	Research at the Willow Creek Ameriflux core site is provided by the Dept. Of Energy Office of Science to the ChEAS Cluster
US-Whs	luckyhills	Walnut Gulch, Lucky Hills Shrubland, Arizona	31.7439	-110.0520	SH	N	Research at Walnut Gulch Experimental Watershed is funded by the USDA-ARS. The Lucky Hills Ameriflux core site is also supported by the Dept. Of Energy Office of Science.
US-Wjs	junipersavannah	Juniper Savannah Site, near Willard, NM	34.4254	-105.8615	EB		AmeriFlux
US-Wkg	kendall	Kendall Grassland, Arizona	31.7365	-109.9419	GR	N	Research at Walnut Gulch Experimental Watershed is funded by the USDA-ARS. The Kendall Ameriflux core site is also supported by the Dept. Of Energy Office of Science.
US-xAB	NEON.D16.ABBY.DP1.00033	NEON Site - D16 (Pacific Northwest) Abby Road, Washington - tower top	45.7624	-122.3303	EN	N	The NEON Data Usage and Citation Policy can be found at: http://data.neonscience.org/data-policy. NEON is a project sponsored by the National Science Foundation and operated under cooperative agreement by Battelle.
	NEON.D16.ABBY.DP1.00042	NEON Site - D16 (Pacific Northwest) Abby Road, Washington - mid-tower	45.7624	-122.3303	EN	N	The NEON Data Usage and Citation Policy can be found at: http://data.neonscience.org/data-policy. NEON is a project sponsored by the National Science Foundation and operated under cooperative agreement by Battelle
US-xAE	NEON.D11.OAES.DP1.00033	NEON Site - D11 (Southern Plains) Klemme Range Research Station, Oklahoma - top-of-tower camera	35.4106	-99.0588	GR	N	The NEON Data Usage and Citation Policy can be found at: http://data.neonscience.org/data-policy. NEON is a project sponsored by the National Science Foundation and operated under cooperative agreement by Battelle.
	NEON.D11.OAES.DP1.00042	NEON Site - D11 (Southern Plains) Klemme Range Research Station, Oklahoma - mid-tower camera	35.4106	-99.0588	GR	N	The NEON Data Usage and Citation Policy can be found at: http://data.neonscience.org/data-policy. NEON is a project sponsored by the National Science Foundation and operated under cooperative agreement by Battelle.
US-xBL	NEON.D02.BLAN.DP1.00033	NEON Site - D02 (Mid-Atlantic) Blandy Experimental Farm, Virginia - tower top	39.0337	-78.0418	DB	N	The NEON Data Usage and Citation Policy can be found at: http://data.neonscience.org/data-policy. NEON is a project sponsored by the National Science Foundation and operated under cooperative agreement by Battelle.
US-xBN	NEON.D19.BONA.DP1.00033	NEON Site - D19 (Taiga) Caribou Creek - Poker Flats Watershed, Alaska - top-of-tower camera	65.1540	-147.5026	EN	N	The NEON Data Usage and Citation Policy can be found at: http://data.neonscience.org/data-policy. NEON is a project sponsored by the National Science Foundation and operated under cooperative agreement by Battelle.

AmeriFlux Code	Camera name	Full site name	Latitude (°)	Longitude (°)	Primary Veg.	Camera Orient.	Acknowledgements
	NEON.D19.BONA.DP1.00042	NEON Site - D19 (Taiga) Caribou Creek - Poker Flats Watershed, Alaska - mid-tower camera	65.1540	-147.5026	EN	N	The NEON Data Usage and Citation Policy can be found at: http://data.neonscience.org/data-policy. NEON is a project sponsored by the National Science Foundation and operated under cooperative agreement by Battelle.
US-xBR	bartlett	Bartlett Experimental Forest, Bartlett, New Hampshire	44.0646	-71.2881	DB	N	Research at the Bartlett Experimental Forest tower is supported by the National Science Foundation (grant DEB-1114804) and the USDA Forest Service's Northern Research Station.
	bartlettir	Bartlett Experimental Forest, Bartlett, New Hampshire	44.0646	-71.2881	DB	N	Research at the Bartlett Experimental Forest tower is supported by the National Science Foundation (grant DEB-1114804) and the USDA Forest Service's Northern Research Station.
	bbc7	Bartlett Experimental Forest, Bartlett, New Hampshire	44.0646	-71.2881	DB	N	
	NEON.D01.BART.DP1.00033	NEON Site - D01 (Northeast) Bartlett Experimental Forest, New Hampshire - top-of-tower camera	44.0639	-71.2874	DB	N	The NEON Data Usage and Citation Policy can be found at: http://data.neonscience.org/data-policy. NEON is a project sponsored by the National Science Foundation and operated under cooperative agreement by Battelle.
	NEON.D01.BART.DP1.00042	NEON Site - D01 (Northeast) Bartlett Experimental Forest, New Hampshire - mid-tower	44.0639	-71.2874	DB	N	The NEON Data Usage and Citation Policy can be found at: http://data.neonscience.org/data-policy. NEON is a project sponsored by the National Science Foundation and operated under cooperative agreement by Battelle.
US-xCL	NEON.D11.CLBJ.DP1.00033	NEON Site - D11 (Southern Plains) LBJ National Grassland, Texas - tower top	33.4012	-97.5700	DB	N	The NEON Data Usage and Citation Policy can be found at: http://data.neonscience.org/data-policy. NEON is a project sponsored by the National Science Foundation and operated under cooperative agreement by Battelle.
	NEON.D11.CLBJ.DP1.00042	NEON Site - D11 (Southern Plains) LBJ National Grassland, Texas - mid-tower	33.4012	-97.5700	DB	N	The NEON Data Usage and Citation Policy can be found at: http://data.neonscience.org/data-policy. NEON is a project sponsored by the National Science Foundation and operated under cooperative agreement by Battelle.
US-xCP	cperagm	CPER Adaptive Grazing Management	40.8402	-104.7672	AG		
	cperheavy	CPER Heavy Grazing Management	40.8153	-104.7386	GR	NW	
	cpertgm	CPER Traditional Grazing Management	40.8330	-104.7600	AG		
	cperuvb	Central Plains Experimental Range, Nunn, Colorado	40.8056	-104.7559	GR	N	Research at the cperuvb site is supported by Colorado State University and the AmericaView program (grants G13AC00393, G11AC20461, G15AC00056) with phenocam equipment and deployment sponsored by the Department of Interior North Central Climate Science Center.
	NEON.D10.CPER.DP1.00033	NEON Site - D10 (Central Plains) Central Plains Experimental Range, Nunn, CO top	40.8155	-104.7456	GR	N	The NEON Data Usage and Citation Policy can be found at: http://data.neonscience.org/data-policy. NEON is a project sponsored by the National Science Foundation and operated under cooperative agreement by Battelle
US-xDC	NEON.D09.DCFS.DP1.00033	NEON Site - D09 (Northern Plains) Dakota Coteau Field School, North Dakota - top-of-tower camera	47.1616	-99.1066	GR	N	The NEON Data Usage and Citation Policy can be found at: http://data.neonscience.org/data-policy. NEON is a project sponsored by the National Science Foundation and operated under cooperative agreement by Battelle.
US-xDC	NEON.D09.DCFS.DP1.00042	NEON Site - D09 (Northern Plains) Dakota Coteau Field School, North Dakota - mid-tower camera	47.1616	-99.1066	GR	N	The NEON Data Usage and Citation Policy can be found at: http://data.neonscience.org/data-policy. NEON is a project sponsored by the National Science Foundation and operated under cooperative agreement by Battelle.
	NEON.D09.PRLA.DP1.20002	NEON Site - D09 (Northern Plains) Prairie Lake at Dakota Coteau Field School, North Dakota - aquatic/stream-gauge camera	47.1591	-99.1139			The NEON Data Usage and Citation Policy can be found at: http://data.neonscience.org/data-policy. NEON is a project sponsored by the National Science Foundation and operated under cooperative agreement by Battelle.
US-xDJ	NEON.D19.DEJU.DP1.00033	NEON Site - D19 (Taiga) Delta Junction, Alaska - tower top	63.8811	-145.7514	EN	N	The NEON Data Usage and Citation Policy can be found at: http://data.neonscience.org/data-policy. NEON is a project sponsored by the National Science Foundation and operated under cooperative agreement by Battelle.
	NEON.D19.DEJU.DP1.00042	NEON Site - D19 (Taiga) Delta Junction, Alaska - mid-tower	63.8811	-145.7514	EN	N	The NEON Data Usage and Citation Policy can be found at: http://data.neonscience.org/data-policy. NEON is a project sponsored by the National Science Foundation and operated under cooperative agreement by Battelle.
US-xDL	NEON.D08.DELA.DP1.00033	NEON Site - D08 (Ozarks Complex) Dead Lake, Alabama - tower top	32.5417	-87.8039	DB	N	The NEON Data Usage and Citation Policy can be found at: http://data.neonscience.org/data-policy. NEON is a project sponsored by the National Science Foundation and operated under cooperative agreement by Battelle.
	NEON.D08.DELA.DP1.00042	NEON Site - D08 (Ozarks Complex) Dead Lake, Alabama - mid-tower	32.5417	-87.8039	DB	N	The NEON Data Usage and Citation Policy can be found at: http://data.neonscience.org/data-policy. NEON is a project sponsored by the National Science Foundation and operated under cooperative agreement by Battelle.
US-xDS	NEON.D03.DSNY.DP1.00033	NEON Site - D03 (Southeast) Disney Wilderness Preserve, Florida - tower top	28.1251	-81.4362	GR	NNE	The NEON Data Usage and Citation Policy can be found at: http://data.neonscience.org/data-policy. NEON is a project sponsored by the National Science Foundation and operated under cooperative agreement by Battelle.
	NEON.D03.DSNY.DP1.00042	NEON Site - D03 (Southeast) Disney Wilderness Preserve, Florida - mid-tower	28.1251	-81.4362	GR	NNE	The NEON Data Usage and Citation Policy can be found at: http://data.neonscience.org/data-policy. NEON is a project sponsored by the National Science Foundation and operated under cooperative agreement by Battelle.
US-xGR	NEON.D07.GRSM.DP1.00033	NEON Site - D07 (Appalachians and Cumberland Plateau) Great Smoky Mountains National Park, Tennessee - tower top	35.6890	-83.5020	DB	N	The NEON Data Usage and Citation Policy can be found at: http://data.neonscience.org/data-policy. NEON is a project sponsored by the National Science Foundation and operated under cooperative agreement by Battelle.
	NEON.D07.GRSM.DP1.00042	NEON Site - D07 (Appalachians and Cumberland Plateau) Great Smoky Mountains National Park, Tennessee - mid-tower	35.6890	-83.5020	DB	N	The NEON Data Usage and Citation Policy can be found at: http://data.neonscience.org/data-policy. NEON is a project sponsored by the National Science Foundation and operated under cooperative agreement by Battelle.
US-xHE	NEON.D19.HEAL.DP1.00033	NEON Site - D19 (Taiga) Healy - top of tower	63.8757	-149.2133	TN	N	
	NEON.D19.HEAL.DP1.00042	NEON Site - D19 (Taiga) Healy - mid-tower	63.8757	-149.2133	TN	N	
US-xJE	NEON.D03.JERC.DP1.00033	NEON Site - D03 (Southeast) Jones Ecological Research Center, Georgia - tower top	31.1948	-84.4686	MX	N	The NEON Data Usage and Citation Policy can be found at: http://data.neonscience.org/data-policy. NEON is a project sponsored by the National Science Foundation and operated under cooperative agreement by Battelle.
	NEON.D03.JERC.DP1.00042	NEON Site - D03 (Southeast) Jones Ecological Research Center, Georgia - mid-tower	31.1948	-84.4686	MX	N	The NEON Data Usage and Citation Policy can be found at: http://data.neonscience.org/data-policy. NEON is a project sponsored by the National Science Foundation and operated under cooperative agreement by Battelle.
US-xJR	ibp	Jornada Experimental Range, New Mexico	32.5890	-106.8470	GR	N	This research at the Jornada Experimental Range is funded by the USDA-Agriculture Research Service (ARS) via ARS Project Number 3050-11210-007-00D. Select camera locations are co-located with sites funded by National Science Foundation under Grant number DEB 1235828 as part of the Jornada Basin LTER program to New Mexico State University.
	ibp0	IBPE-1 Camera, Jornada Experimental Range, New Mexico	32.5890	-106.8470	GR	N	This research at the Jornada Experimental Range is funded by the USDA-Agriculture Research Service (ARS) via ARS Project Number 3050-11210-007-00D. Select camera locations are co-located with sites funded by National

AmeriFlux Code	Camera name	Full site name	Latitude (°)	Longitude (°)	Primary Veg.	Camera Orient.	Acknowledgements
							Science Foundation under Grant number DEB 1235828 as part of the Jornada Basin LTER program to New Mexico State University.
	jergrassland	Black grama grassland at Jornada Experimental Range	32.5833	-106.8333	GR	N	USDA Agricultural Research Service
	jergrassland2	Black grama grassland at Jornada Experimental Range	32.5849	-106.8261	GR	N	This research was a contribution from the Long-Term Agroecosystem Research (LTAR) network. LTAR is supported by the United States Department of Agriculture.
	jernovel	Grass-recovered shrubland on Jornada Experimental Range	32.6335	-106.8334	SH	N	USDA Agricultural Research Service LTAR network
	NEON.D14.JORN.DP1.00033	NEON Site - D14 (Desert Southwest) Jornada LTER, New Mexico - top-of-tower camera	32.5907	-106.8425	GR	N	The NEON Data Usage and Citation Policy can be found at: http://data.neonscience.org/data-policy. NEON is a project sponsored by the National Science Foundation and operated under cooperative agreement by Battelle.
	NEON.D14.JORN.DP1.00042	NEON Site - D14 (Desert Southwest) Jornada LTER, New Mexico - mid-tower camera	32.5907	-106.8425	GR	N	The NEON Data Usage and Citation Policy can be found at: http://data.neonscience.org/data-policy. NEON is a project sponsored by the National Science Foundation and operated under cooperative agreement by Battelle.
US-xKA/ US-xKZ	konza	Konza Prairie Biological Station, Kansas State University, Kansas	39.0824	-96.5603	GR	NW	The US-Kon site acknowledges support from the LTER program at the Konza Prairie Biological Station (DEB-0823341), and the U.S. Department of Energy under a subcontract from DE-AC02-05CH11231.
US-xKA/ US-xKZ	NEON.D06.KING.DP1.20002	NEON Site - D06 (Prairie Peninsula) Kings Creek, Kansas - aquatic/stream-gauge camera	39.1051	-96.6034			The NEON Data Usage and Citation Policy can be found at: http://data.neonscience.org/data-policy. NEON is a project sponsored by the National Science Foundation and operated under cooperative agreement by Battelle.
	NEON.D06.KONA.DP1.00033	NEON Site - D06 (Prairie Peninsula) Konza Prairie Biological Station - top	39.1104	-96.6129	AG	N	
	NEON.D06.KONA.DP1.00042	NEON Site - D06 (Prairie Peninsula) Konza Prairie Biological Station - mid	39.1104	-96.6129	AG	N	
	NEON.D06.KONZ.DP1.00033	NEON Site - D06 (Prairie Peninsula) Konza Prairie Biological Station, Kansas - top-of-tower camera	39.1008	-96.5631	GR	N	The NEON Data Usage and Citation Policy can be found at: http://data.neonscience.org/data-policy. NEON is a project sponsored by the National Science Foundation and operated under cooperative agreement by Battelle.
	NEON.D06.KONZ.DP1.00042	NEON Site - D06 (Prairie Peninsula) Konza Prairie Biological Station, Kansas - mid-tower camera	39.1008	-96.5631	GR	N	The NEON Data Usage and Citation Policy can be found at: http://data.neonscience.org/data-policy. NEON is a project sponsored by the National Science Foundation and operated under cooperative agreement by Battelle.
US-xLE	NEON.D08.LENO.DP1.00033	NEON Site - D08 (Ozarks Complex) Lenoir Landing, AL - top of tower	31.8539	-88.1612	DB	N	
	NEON.D08.LENO.DP1.00042	NEON Site - D08 (Ozarks Complex) Lenoir Landing, AL - mid tower	31.8539	-88.1612	DB	N	
	NEON.D08.TOMB.DP1.20002	NEON Site - D08 (Ozarks Complex) Tombigbee River, Alabama - aquatic/stream-gauge camera	31.8534	-88.1589			The NEON Data Usage and Citation Policy can be found at: http://data.neonscience.org/data-policy. NEON is a project sponsored by the National Science Foundation and operated under cooperative agreement by Battelle.
US-xMB	NEON.D13.MOAB.DP1.00033	NEON Site - D13 (Southern Rockies and Colorado Plateau) Moab, Utah - top-of-tower camera	38.2483	-109.3883	GR	N	The NEON Data Usage and Citation Policy can be found at: http://data.neonscience.org/data-policy. NEON is a project sponsored by the National Science Foundation and operated under cooperative agreement by Battelle.
	NEON.D13.MOAB.DP1.00042	NEON Site - D13 (Southern Rockies and Colorado Plateau) Moab, Utah - mid-tower camera	38.2483	-109.3883	GR	N	The NEON Data Usage and Citation Policy can be found at: http://data.neonscience.org/data-policy. NEON is a project sponsored by the National Science Foundation and operated under cooperative agreement by Battelle.
US-xML	NEON.D07.MLBS.DP1.00033	NEON Site - D07 (Appalachians and Cumberland Plateau) Mountain Lake Biological Station, Virginia - tower top	37.3783	-80.5248	DB	N	The NEON Data Usage and Citation Policy can be found at: http://data.neonscience.org/data-policy. NEON is a project sponsored by the National Science Foundation and operated under cooperative agreement by Battelle.
	NEON.D07.MLBS.DP1.00042	NEON Site - D07 (Appalachians and Cumberland Plateau) Mountain Lake Biological Station, Virginia - mid-tower	37.3783	-80.5248	DB	N	The NEON Data Usage and Citation Policy can be found at: http://data.neonscience.org/data-policy. NEON is a project sponsored by the National Science Foundation and operated under cooperative agreement by Battelle.
US-xNG	mandanh5	Dryland Cropping System, Mandan, North Dakota	46.7754	-100.9511	AG	N	Research at the Mandan site is funded by the USDA-Agricultural Research Service (ARS) via ARS Project Number 3064-21660-003-00, in coordination with the Long-term Agroecosystem Research (LTAR) network. Site availability is facilitated by the Area IV Soil Conservation Districts in North Dakota.
	mandani2	Dryland Cropping System, Mandan, North Dakota	46.7614	-100.9257	AG	N	Research at the Mandan site is funded by the USDA-Agricultural Research Service (ARS) via ARS Project Number 3064-21660-003-00, in coordination with the Long-term Agroecosystem Research (LTAR) network. Site availability is facilitated by the Area IV Soil Conservation Districts in North Dakota.
	NEON.D09.NOGP.DP1.00033	NEON Site - D09 (Northern Plains) Northern Great Plains Research Laboratory, North Dakota - top-of-tower camera	46.7697	-100.9154	GR	N	The NEON Data Usage and Citation Policy can be found at: http://data.neonscience.org/data-policy. NEON is a project sponsored by the National Science Foundation and operated under cooperative agreement by Battelle.
	NEON.D09.NOGP.DP1.00042	NEON Site - D09 (Northern Plains) Northern Great Plains Research Laboratory, North Dakota - mid-tower camera	46.7697	-100.9154	GR	N	The NEON Data Usage and Citation Policy can be found at: http://data.neonscience.org/data-policy. NEON is a project sponsored by the National Science Foundation and operated under cooperative agreement by Battelle.
US-xNQ	NEON.D15.ONAQ.DP1.00033	NEON Site - D15 (Great Basin) Onaqui, Utah - top-of-tower camera	40.1776	-112.4525	SH	NNE	The NEON Data Usage and Citation Policy can be found at: http://data.neonscience.org/data-policy. NEON is a project sponsored by the National Science Foundation and operated under cooperative agreement by Battelle.
	NEON.D15.ONAQ.DP1.00042	NEON Site - D15 (Great Basin) Onaqui, Utah - mid-tower camera	40.1776	-112.4525	SH	NNE	The NEON Data Usage and Citation Policy can be found at: http://data.neonscience.org/data-policy. NEON is a project sponsored by the National Science Foundation and operated under cooperative agreement by Battelle.
US-xPU	NEON.D20.PUUM.DP1.00033	NEON Site - D20 (Pacific Tropical) Pu`u Maka`ala Natural Area Reserve, Hawaii - top-of-tower camera	19.5531	-155.3173	EB		The NEON Data Usage and Citation Policy can be found at: http://data.neonscience.org/data-policy. NEON is a project sponsored by the National Science Foundation and operated under cooperative agreement by Battelle.
	NEON.D20.PUUM.DP1.00042	NEON Site - D20 (Pacific Tropical) Pu`u Maka`ala Natural Area Reserve, Hawaii - mid-tower camera	19.5531	-155.3173	EB		The NEON Data Usage and Citation Policy can be found at: http://data.neonscience.org/data-policy. NEON is a project sponsored by the National Science Foundation and operated under cooperative agreement by Battelle.
US-xRM	NEON.D10.RMNP.DP1.00033	NEON Site - D10 (Central Plains) Rocky Mountain National Park CASTNET, Colorado - tower top	40.2759	-105.5460	EN	N	The NEON Data Usage and Citation Policy can be found at: http://data.neonscience.org/data-policy. NEON is a project sponsored by the National Science Foundation and operated under cooperative agreement by Battelle.
US-xRN	NEON.D07.ORNL.DP1.00033	NEON Site - D07 (Appalachians and Cumberland Plateau) Oak Ridge, Tennessee - tower top	35.9641	-84.2826	DB	W	The NEON Data Usage and Citation Policy can be found at: http://data.neonscience.org/data-policy. NEON is a project sponsored by the National Science Foundation and operated under cooperative agreement by Battelle.
	NEON.D07.ORNL.DP1.00042	NEON Site - D07 (Appalachians and Cumberland Plateau) Oak Ridge, Tennessee - mid-tower	35.9641	-84.2826	DB	W	The NEON Data Usage and Citation Policy can be found at: http://data.neonscience.org/data-policy. NEON is a project sponsored by the National Science Foundation and operated under cooperative agreement by Battelle.

AmeriFlux Code	Camera name	Full site name	Latitude (°)	Longitude (°)	Primary Veg.	Camera Orient.	Acknowledgements
	NEON.D07.WALK.DP1.20002	NEON Site - D07 (Appalachians & Cumberland Plateau) Walker Ranch, TN	35.9595	-84.2804			
	oakridge1	Chestnut Ridge, Oak Ridge, Tennessee	35.9311	-84.3323	DB	N	Research at Chestnut Ridge is funded by US Dept of Commerce, National Oceanic and Atmospheric Administration, Office of Atmospheric Research, Air Resources Lab, Atmospheric Turbulence and Diffusion Division as part of the Surface Energy Budget Network (SEBN)
	oakridge2	Chestnut Ridge, Oak Ridge, Tennessee	35.9311	-84.3323	DB	SW	Research at Chestnut Ridge is funded by US Dept of Commerce, National Oceanic and Atmospheric Administration, Office of Atmospheric Research, Air Resources Lab, Atmospheric Turbulence and Diffusion Division as part of the Surface Energy Budget Network (SEBN)
	NEON.D03.BARC.DP1.20002	NEON Site - D03 (Southeast) Ordway-Swisher Biological Station - Barco Lake, Florida -	29.6765	-82.0091			The NEON Data Usage and Citation Policy can be found at: http://data.neonscience.org/data-policy. NEON is a project sponsored by the National Science Foundation and operated under cooperative agreement by Battelle.
US-xSB	NEON.D03.OSBS.DP1.00033	NEON Site - D03 (Southeast) Ordway-Swisher Biological Station, Florida - tower top	29.6893	-81.9934	EN	N	The NEON Data Usage and Citation Policy can be found at: http://data.neonscience.org/data-policy. NEON is a project sponsored by the National Science Foundation and operated under cooperative agreement by Battelle.
	NEON.D03.OSBS.DP1.00042	NEON Site - D03 (Southeast) Ordway-Swisher Biological Station, Florida - mid-tower	29.6893	-81.9934	EN	N	The NEON Data Usage and Citation Policy can be found at: http://data.neonscience.org/data-policy. NEON is a project sponsored by the National Science Foundation and operated under cooperative agreement by Battelle.
US-xSC	NEON.D02.SCBI.DP1.00033	NEON Site - D02 (Mid-Atlantic) Smithsonian Conservation Biology Institute, Virginia - tower top	38.8929	-78.1395	DB	N	The NEON Data Usage and Citation Policy can be found at: http://data.neonscience.org/data-policy. NEON is a project sponsored by the National Science Foundation and operated under cooperative agreement by Battelle.
US-xSE	NEON.D02.SERC.DP1.00033	NEON Site - D02 (Mid-Atlantic) Smithsonian Environmental Research Center, top	38.8901	-76.5600	DB	N	
	NEON.D02.SERC.DP1.00042	NEON Site - D02 (Mid-Atlantic) Smithsonian Environmental Research Center, mid	38.8901	-76.5600	DB	N	
US-xSJ	NEON.D17.SJER.DP1.00033	NEON Site - D17 (Pacific Southwest) San Joaquin, California - top-of-tower camera	37.1088	-119.7323	EN		The NEON Data Usage and Citation Policy can be found at: http://data.neonscience.org/data-policy. NEON is a project sponsored by the National Science Foundation and operated under cooperative agreement by Battelle.
	NEON.D17.SJER.DP1.00042	NEON Site - D17 (Pacific Southwest) San Joaquin, California - mid-tower camera	37.1088	-119.7323	EN		The NEON Data Usage and Citation Policy can be found at: http://data.neonscience.org/data-policy. NEON is a project sponsored by the National Science Foundation and operated under cooperative agreement by Battelle.
US-xSL	NEON.D10.STER.DP1.00033	NEON Site - D10 (Central Plains) Sterling, Colorado - tower top	40.4619	-103.0293	AG	N	The NEON Data Usage and Citation Policy can be found at: http://data.neonscience.org/data-policy. NEON is a project sponsored by the National Science Foundation and operated under cooperative agreement by Battelle.
US-xSP	NEON.D17.SOAP.DP1.00033	NEON Site - D17 (Pacific Southwest) Soaproot Saddle, California - top-of-tower camera	37.0334	-119.2622	EN	N	The NEON Data Usage and Citation Policy can be found at: http://data.neonscience.org/data-policy. NEON is a project sponsored by the National Science Foundation and operated under cooperative agreement by Battelle.
	NEON.D17.SOAP.DP1.00042	NEON Site - D17 (Pacific Southwest) Soaproot Saddle, California - mid-tower camera	37.0334	-119.2622	EN	N	The NEON Data Usage and Citation Policy can be found at: http://data.neonscience.org/data-policy. NEON is a project sponsored by the National Science Foundation and operated under cooperative agreement by Battelle.
US-xSR	NEON.D14.SRER.DP1.00033	NEON Site - D14 (Desert Southwest) Santa Rita Experimental Range, Arizona - tower top	31.9107	-110.8355	SH	N	The NEON Data Usage and Citation Policy can be found at: http://data.neonscience.org/data-policy. NEON is a project sponsored by the National Science Foundation and operated under cooperative agreement by Battelle.
	NEON.D14.SRER.DP1.00042	NEON Site - D14 (Desert Southwest) Santa Rita Experimental Range, Arizona - mid-tower	31.9107	-110.8355	SH	N	The NEON Data Usage and Citation Policy can be found at: http://data.neonscience.org/data-policy. NEON is a project sponsored by the National Science Foundation and operated under cooperative agreement by Battelle.
US-xST/ US-xTR	NEON.D05.STEI.DP1.00033	NEON Site - D05 (Great Lakes) Steigerwaldt Land Services, Wisconsin - top-of-tower camera	45.5089	-89.5864	DB	N	The NEON Data Usage and Citation Policy can be found at: http://data.neonscience.org/data-policy. NEON is a project sponsored by the National Science Foundation and operated under cooperative agreement by Battelle.
	NEON.D05.STEI.DP1.00042	NEON Site - D05 (Great Lakes) Steigerwaldt Land Services, Wisconsin - mid-tower camera	45.5089	-89.5864	DB	N	The NEON Data Usage and Citation Policy can be found at: http://data.neonscience.org/data-policy. NEON is a project sponsored by the National Science Foundation and operated under cooperative agreement by Battelle.
US-xST/ US-xTR	NEON.D05.TREE.DP1.00033	NEON Site - D05 (Great Lakes) Treehaven, Wisconsin - tower top	45.4937	-89.5857	MX	N	The NEON Data Usage and Citation Policy can be found at: http://data.neonscience.org/data-policy. NEON is a project sponsored by the National Science Foundation and operated under cooperative agreement by Battelle.
	NEON.D05.TREE.DP1.00042	NEON Site - D05 (Great Lakes) Treehaven, Wisconsin - mid-tower	45.4937	-89.5857	MX	N	The NEON Data Usage and Citation Policy can be found at: http://data.neonscience.org/data-policy. NEON is a project sponsored by the National Science Foundation and operated under cooperative agreement by Battelle.
US-xTA	NEON.D08.TALL.DP1.00033	NEON Site - D08 (Ozarks Complex) Talladega National Forest, Alabama - tower top	32.9505	-87.3933	EN	N	The NEON Data Usage and Citation Policy can be found at: http://data.neonscience.org/data-policy. NEON is a project sponsored by the National Science Foundation and operated under cooperative agreement by Battelle.
	NEON.D08.TALL.DP1.00042	NEON Site - D08 (Ozarks Complex) Talladega National Forest, Alabama - mid-tower	32.9505	-87.3933	EN	N	The NEON Data Usage and Citation Policy can be found at: http://data.neonscience.org/data-policy. NEON is a project sponsored by the National Science Foundation and operated under cooperative agreement by Battelle.
US-xTE	NEON.D17.TEAK.DP1.00033	NEON Site - D17 (Pacific Southwest) Lower Teakettle, California - top-of-tower camera	37.0058	-119.0060	EN		The NEON Data Usage and Citation Policy can be found at: http://data.neonscience.org/data-policy. NEON is a project sponsored by the National Science Foundation and operated under cooperative agreement by Battelle.
	NEON.D17.TEAK.DP1.00042	NEON Site - D17 (Pacific Southwest) Lower Teakettle, California - mid-tower camera	37.0058	-119.0060	EN		The NEON Data Usage and Citation Policy can be found at: http://data.neonscience.org/data-policy. NEON is a project sponsored by the National Science Foundation and operated under cooperative agreement by Battelle.
US-xTL	NEON.D18.TOOL.DP1.00033	NEON Site - D18 (Tundra) Toolik, Alaska - tower top	68.6611	-149.3705	TN	N	The NEON Data Usage and Citation Policy can be found at: http://data.neonscience.org/data-policy. NEON is a project sponsored by the National Science Foundation and operated under cooperative agreement by Battelle.
	NEON.D18.TOOL.DP1.00042	NEON Site - D18 (Tundra) Toolik, Alaska - mid-tower	68.6611	-149.3705	TN	N	The NEON Data Usage and Citation Policy can be found at: http://data.neonscience.org/data-policy. NEON is a project sponsored by the National Science Foundation and operated under cooperative agreement by Battelle.
	NEON.D05.CRAM.DP1.20002	NEON Site - D05 (Great Lakes) Crampton Lake, Wisconsin - aquatic/stream-gauge camera	46.2111	-89.4783			The NEON Data Usage and Citation Policy can be found at: http://data.neonscience.org/data-policy. NEON is a project sponsored by the National Science Foundation and operated under cooperative agreement by Battelle.
US-xUN	NEON.D05.UNDE.DP1.00033	NEON Site - D05 (Great Lakes) UNDERC, Michigan - tower top	46.2339	-89.5373	DB	N	The NEON Data Usage and Citation Policy can be found at: http://data.neonscience.org/data-policy. NEON is a project sponsored by the National Science Foundation and operated under cooperative agreement by Battelle.
	NEON.D05.UNDE.DP1.00042	NEON Site - D05 (Great Lakes) UNDERC, Michigan - mid-tower	46.2339	-89.5373	DB	N	The NEON Data Usage and Citation Policy can be found at: http://data.neonscience.org/data-policy. NEON is a project sponsored by the National Science Foundation and operated under cooperative agreement by Battelle.

AmeriFlux Code	Camera name	Full site name	Latitude (°)	Longitude (°)	Primary Veg.	Camera Orient.	Acknowledgements
US-xWD	NEON.D09.PRPO.DP1.20002	NEON Site - D09 (Northern Plains) Prairie Pothole, North Dakota -	47.1300	-99.2506			The NEON Data Usage and Citation Policy can be found at: http://data.neonscience.org/data-policy. NEON is a project sponsored by the National Science Foundation and operated under cooperative agreement by Battelle.
	NEON.D09.WOOD.DP1.00033	NEON Site - D09 (Northern Plains) Woodworth, North Dakota - tower top	47.1282	-99.2413	GR	N	The NEON Data Usage and Citation Policy can be found at: http://data.neonscience.org/data-policy. NEON is a project sponsored by the National Science Foundation and operated under cooperative agreement by Battelle.
US-xWR	NEON.D16.WREF.DP1.00033	NEON Site - D16 (Pacific Northwest) Wind River Experimental Forest, Washington - top-of-tower camera	45.8205	-121.9519	EN	N	The NEON Data Usage and Citation Policy can be found at: http://data.neonscience.org/data-policy. NEON is a project sponsored by the National Science Foundation and operated under cooperative agreement by Battelle.
	NEON.D16.WREF.DP1.00042	NEON Site - D16 (Pacific Northwest) Wind River Experimental Forest, Washington - mid-tower camera	45.8205	-121.9519	EN	N	The NEON Data Usage and Citation Policy can be found at: http://data.neonscience.org/data-policy. NEON is a project sponsored by the National Science Foundation and operated under cooperative agreement by Battelle.
	windriver	Thornton T. Munger Research Natural Area, Wind River Experimental Forest, Washington	45.8213	-121.9521	EN	N	Data and logistical support were provided by the US Forest Service Pacific Northwest Research Station and the University of Washington
	windriverunderstory	Understory Camera, Thornton T. Munger Research Natural Area, Wind River Experimental Forest, Washington	45.8213	-121.9521	EN	N	Data and logistical support were provided by the Wind River Field Station, a joint scientific venture financed by the Pacific Northwest Research Station and the University of Washington
US-xYE	NEON.D12.BLDE.DP1.20002	NEON Site - D12 (Northern Rockies) Blacktail Deer Creek, Wyoming - aquatic/stream-gauge camera	44.9501	-110.5872			The NEON Data Usage and Citation Policy can be found at: http://data.neonscience.org/data-policy. NEON is a project sponsored by the National Science Foundation and operated under cooperative agreement by Battelle.
	NEON.D12.YELL.DP1.00033	NEON Site - D12 (Northern Rockies) Yellowstone National Park, Wyoming - top-of-tower camera	44.9535	-110.5391	EN		The NEON Data Usage and Citation Policy can be found at: http://data.neonscience.org/data-policy. NEON is a project sponsored by the National Science Foundation and operated under cooperative agreement by Battelle.
	NEON.D12.YELL.DP1.00042	NEON Site - D12 (Northern Rockies) Yellowstone National Park, Wyoming - mid-tower camera	44.9535	-110.5391	EN		The NEON Data Usage and Citation Policy can be found at: http://data.neonscience.org/data-policy. NEON is a project sponsored by the National Science Foundation and operated under cooperative agreement by Battelle.
